# When Language Background Does Not Matter: Both Mono‐ and Bilingual Children Use Mutual Exclusivity and Pragmatic Context to Learn Novel Words

**DOI:** 10.1111/desc.13618

**Published:** 2025-03-11

**Authors:** Natalie Bleijlevens, Anna‐Lena Ciesla, Tanya Behne

**Affiliations:** ^1^ Department of Developmental Psychology University of Göttingen Göttingen Germany; ^2^ Leibniz ScienceCampus “Primate Cognition” Göttingen Germany

**Keywords:** bilingualism, common ground, disambiguation, mutual exclusivity, pragmatics, word learning

## Abstract

Do mono‐ and bilingual children differ in the way they learn novel words in ambiguous settings? Listeners may resolve referential ambiguity by assuming that novel words refer to unknown, rather than known, objects–a response known as the *mutual exclusivity effect*. Past research suggested that mono‐ and bilinguals differ with regard to this disambiguation strategy, perhaps because, across languages, bilinguals’ experience contradicts one‐to‐one mappings of label and referent. Another line of research suggested a bilingual advantage in resolving referential ambiguity, based on bilinguals’ advanced pragmatic skills. Here, we examine both these claims in a preregistered study with comparable samples of mono‐ and bilingual 3‐year‐olds (*n* = 74) and adults (*n* = 86). We tested referent disambiguation and retention in two tasks: In the Mutual‐Exclusivity task, a speaker used a novel label in the presence of a known and an unknown object. In the Pragmatic task, she used another novel label in the presence of two unknown objects and participants could infer from the pragmatic context that the speaker referred to the object that was new in their discourse. Mono‐ and bilinguals were equally successful in inferring the correct label‐referent links in both tasks and retained them after a delay. These findings indicate that children with different language backgrounds can develop the same strategies and pragmatic skills to learn novel words. Children can use their lexical knowledge and socio‐cognitive skills to infer the meanings of novel words, irrespective of whether they are acquiring one or more languages.

## 1 Introduction

Young language learners face many challenges. Based on limited vocabulary and language experience, they need to find out what their conversation partner is referring to by using a novel word. Crucially, they often need to do so while facing referential ambiguity, that is, in the presence of several potential referents. Despite these challenges, children come to learn the words they are exposed to; and they do so regardless of whether they are learning only one or several native languages. Mono‐ and multilingual children differ fundamentally in their language experience—but do they also differ in the strategies they lean on to learn novel word meanings?

**Summary**

- We tested if mono‐ and bilingual preschoolers (and adults) differ in the strategies and pragmatic skills used to disambiguate and learn novel words.
- Both language groups successfully disambiguated and retained words in both a mutual exclusivity task and a task that required interpreting the pragmatic context.
- Mono‐ and bilingual participants did not differ in their performance in either task or in either age group.
- These findings contribute to ongoing theoretical debates about whether (and, if so, why) mono‐ and bilinguals might differ in their word‐learning strategies.

One line of research proposes that mono‐ and bilinguals differ in the use of strategies to disambiguate novel word meanings. For example, listeners may resolve referential ambiguity by assuming that a novel label refers to a novel rather than a name‐known object—a strategy known as the “mutual exclusivity” (ME) effect (see e.g., Lewis et al. 2020). While monolingual children reliably use this strategy, research with bilingual children suggests that they are less likely to do so (e.g., Byers‐Heinlein and Werker 2009). A second line of research proposes that bilinguals have advantages in disambiguating novel word meaning. For example, it has been suggested that the demands of growing up in a bilingual environment result in superior pragmatic abilities that may in turn aid children's referent identification (Siegal et al. 2009; Wermelinger et al. 2017; Yow et al. 2017; Yow and Markman 2015). We will examine these two lines of research in turn.^1^

### 1.1 The Effect of Linguistic Background on Referent Disambiguation by Mutual Exclusivity

Children often need to find the referents of novel words in the light of referential ambiguity, that is, when several potential referents are present. Children, at least monolinguals, have been demonstrated to resolve this ambiguity by showing the mutual exclusivity (ME) effect: They assume that the novel label refers to a novel/label‐unknown, rather than to a familiar/label‐known, object (Lewis et al. 2020; Markman and Wachtel 1988). But why might children's language background, that is, growing up in a mono‐ versus bilingual environment, affect how they respond (e.g., Byers‐Heinlein and Werker 2009)? Why might bilingual children be less likely to show the ME effect? Two different types of explanations have been put forward.

The first is that bilingual children do not show the ME effect because they differ in the specific capability or bias that this word learning strategy is based on. Different theoretical explanations have been proposed to explain the ME effect (Lewis et al. 2020). One influential account, the lexical constraint account, suggests that the ME effect is based on the assumption that objects only have one basic‐level label (known as the *ME bias*; Markman and Wachtel 1988). Thus, when children hear a novel label, they exclude the familiar object as a potential referent, because it already has a label. However, children raised in a bilingual environment constantly experience different labels (across languages) used to refer to the same object and, thus, bilingual children might not show this mutual exclusivity bias (either because they never develop it, or because they discard it again) (e.g., Byers‐Heinlein and Werker 2009; Davidson et al. 1997; Houston‐Price et al. 2010).^2^

The second type of explanation is that the ME strategy may be available to both mono‐ and bilingual children in principle, but bilinguals may not always show the ME effect for various reasons. For example, bilinguals have smaller productive vocabularies in each of their languages compared to their monolingual peers (e.g., Bialystok et al. 2010). If bilinguals’ knowledge of the familiar distractor labels is more fragile, this may result in a weaker ME effect (Grassmann et al. 2015; Lewis et al. 2020). This may be especially relevant in the case of young infants (Byers‐Heinlein and Werker 2009, Lewis et al. 2020). In addition, bilinguals may be less comfortable with the test language than monolinguals, which may influence how they respond in such tests. Thus, while the first type of explanations proposed competence differences between mono‐ and bilingual children (with regard to their word learning strategies), the second type of explanations proposed performance differences due to effects of the test language.

However, irrespective of *how* potential differences in ME performance between monolingual and bilingual children may be explained, the more fundamental question that needs examining is *whether* the reported difference is a robust effect. Do mono‐ and bilingual children really differ in their ME performance? A closer look at the empirical findings reveals a mixed pattern: While some studies find that bilinguals are less likely to show the ME effect than monolinguals (Byers‐Heinlein and Werker 2009; Houston‐Price et al. 2010; Repnik et al. 2021), other studies could not replicate this difference (Byers‐Heinlein et al. 2014; I. Frank and Poulin‐Dubois 2002; Kalashnikova et al. 2015, Kalashnikova et al. 2018; Rocha‐Hidalgo et al. 2021; Rochanavibhata et al. 2022; Weatherhead et al. 2021). A meta‐analysis, including 12 studies with multilingual participants, suggested that the magnitude of the ME effect is influenced by the participants’ language background as well as their age: The effect sizes tended to be larger for monolingual and older children (Lewis et al. 2020). This analysis, however, did not include more recent research (published after 2017)—much of which did not find a difference in ME performance between mono‐ and bilingual children (e.g., Kalashnikova et al. 2018; Rocha‐Hidalgo et al. 2021; Rochanavibhata et al. 2022; Weatherhead et al. 2021).

Some of these recent studies have suggested a new twist on how word learning in ME contexts may differ between mono‐ and bilinguals: They distinguished between the use of ME to identify the intended object in the moment of interaction and the long‐term retention of this label‐object link (Kalashnikova et al. 2018; Repnik et al. 2021; Rocha‐Hidalgo et al. 2021; Weatherhead et al. 2021). This distinction is based on theoretical (e.g., McMurray et al. 2012) and empirical work (e.g., Horst and Samuelson 2008), suggesting that referent disambiguation and retention should not be conflated and that in order to evaluate children's word learning, their retention of the identified label‐object link needs to be assessed after a 5‐min delay (to ensure that word learning was based on a retrieval from long‐term memory, see Horst and Samuelson 2008).

Thus, in addition to children's immediate disambiguation responses, these studies comparing the performance of mono‐ and bilingual children also assessed their retention of the novel label‐referent link after a delay. And while in these studies bilinguals showed the ME effect in their immediate referent disambiguation just as their monolingual peers did, bilingual 2‐year‐olds did not select the targets above chance in subsequent retention trials (Kalashnikova et al. 2018; Repnik et al. 2021; Rocha‐Hidalgo et al. 2021; but see Weatherhead et al. 2021). Specifically, in a study by Kalashnikova et al. (2018), at 18 months of age both mono‐ and bilingual children showed retention of the novel labels they encountered in a ME context. However, at 24 months mono‐ and bilingual children differed significantly in their word‐learning performance: Whereas at 24‐month‐old monolingual children retained the novel labels, their 24‐month‐old bilingual peers did not. This led to the proposal that, with increasing experience with language use, the ME strategy develops into a reliable word‐learning strategy for monolingual but not for bilingual children (Kalashnikova et al. 2018; *cf*. Rocha‐Hidalgo et al. 2021). The idea here is that, based on their experience with many‐to‐one mappings across languages, bilingual toddlers begin to learn that ME is not a reliable word‐*learning* strategy for them and will stop applying it.

To evaluate this proposal regarding this difference in word learning strategies, research with young preschoolers may be especially informative. If it is indeed the case that bilinguals’ experience leads them to discard ME as a basis for word learning from their second birthday onwards, then a difference between mono‐ and bilingual children in their use of the ME strategy should become more pronounced and easily demonstratable in young preschoolers. In other words, bilingual 3‐year‐olds should not show word learning in an ME context. In contrast, if bilinguals use and retain ME as a word learning strategy, but may sometimes show more fragile performance, especially during their first 2 years of life (e.g., due to uncertainty about the familiar distractor label), then difficulties should not persist in preschool years (especially if distractor objects are used whose labels are highly familiar by this age to both mono‐ and bilingual peers). Thus, in this latter case, both mono‐ and bilingual 3‐year‐olds should demonstrate competence in word‐learning based on the ME strategy.

#### 1.1.1 Methodological Considerations

The mixed pattern of findings regarding the effect of language background may reflect methodological issues. One potential issue concerns small and unbalanced sample sizes. Small sample sizes generally come with problems of statistical power. This becomes particularly problematic if the sample sizes are unbalanced (i.e., more mono‐ than bilinguals are tested) or if the reported difference in performance is established by testing each group's performance against chance, with no direct comparison of mono‐ and bilingual samples. Furthermore, since bilinguals, in contrast to monolinguals, are often not tested in their first language, they may feel less comfortable in the test language, causing differences in their responses that are independent of the capacities of interest.

In sum, past research suggested mono‐ and bilinguals differ in how they disambiguate novel word meanings in the ME task and retain these mappings subsequently. However, mixed findings and methodological concerns highlight the need for preregistered research that tests balanced samples of mono‐ and bilingual preschoolers, based on a priori power analyses, to assess the robustness of the suggested effects. Given the suggestion that differences between language groups in novel word retention develop from 2 years onward (see Kalashnikova et al. 2018), the assessment of young preschoolers is especially relevant.

### 1.2 The Effect of Linguistic Background on Pragmatic Skills

A second, more or less independent, line of research has suggested that bilinguals have advantages in other word learning areas. Specifically, bilinguals may outperform their monolingual peers in their pragmatic and social‐communicative skills (e.g., Fan et al. 2015; Yow and Markman 2015). Again, there are different explanations for the potential differences between mono‐ and bilinguals.

First, bilinguals’ language experience may lead to advanced communicative and socio‐cognitive abilities. These advantages may be a consequence of the communicative challenges they are facing in their daily lives—such as communicative failures, misunderstandings, and adapting to an environment using different languages (Fan et al. 2015; Liberman et al. 2017; Wermelinger et al. 2017)—and may even be a way of dealing with an initially smaller vocabulary in each of their languages (Siegal et al. 2009). Bilinguals’ continuous demands to flexibly adjust their linguistic interactions to their conversation partner, may train their communicative skills, as well as their perspective taking (Schroeder 2018) and executive function in general (Ware et al. 2020).

Second, the bilingual pragmatic advantages may also be a consequence of a systematic selection bias. Bilingual populations do not only differ from monolinguals in terms of their language background. Bilingual families often immigrated from another country (and cultural background) and may potentially be more open‐minded and socially sensitive, which may in turn lead to social‐cognitive advantages that are not specifically due to their language experience (see, e.g., Gampe et al. 2020 for an example in which not bilingualism per se, but the cultural background of child and caregiver shapes communicative interactions).

The empirical basis for the proposed bilingual pragmatic advantages is, again, mixed (van Wonderen et al. 2023). Bilingual advantages are mainly found in younger children, and may depend on the specific task or ability in question (see Antoniou et al. 2020). They may be especially pronounced in tasks that tap more basic pragmatic and social‐communicative skills, such as perspective taking (Fan et al. 2015; Liberman et al. 2017) and Theory of Mind in general (Schroeder 2018), understanding referential intent (Yow et al. 2017; Yow and Markman 2015), or repairing communication failures (Wermelinger et al. 2017). Differences may be less robust, however, in more complex pragmatic abilities, such as irony, sarcasm, metaphors or implicatures (Antoniou et al. 2020; Syrett et al. 2017; but see Siegal et al. 2009). Overall, past research on bilingual pragmatic advantages is relatively sparse and it remains open if the proposed advantages are replicable and to which specific pragmatic abilities they apply.

### 1.3 The Current Study

Past research has suggested that mono‐ and bilingual children may differ in the strategies and pragmatic abilities underlying their word learning. However, there is uncertainty regarding the robustness, as well as the explanations for these potential differences. In the current project, we focus on the two contexts in which differences have been suggested: differences between mono‐ and bilinguals with regard to (i) the use of ME to disambiguate and learn novel word‐object links and (ii) the use of social‐pragmatic information for word learning.

Combining both lines of research, we tested the *same* participants in both contexts to assess in a common design whether mono‐ and bilingual children differ in the strategies and pragmatic abilities they use for referent disambiguation and word learning, or whether potential differences may vanish when the tested samples of mono‐ and bilinguals are sufficiently comparable. Testing the same participants in both their use of ME and their use of socio‐pragmatic information for word‐learning is also relevant with regard to theoretical debates on the cognitive bases of the ME response—specifically the debate between lexical constraint accounts and pragmatic accounts. As described above, the lexical constraint accounts propose that the ME response is based on the assumption that each object has only one basic‐level label (Markman and Wachtel 1988). Hence, children's ME response may be independent from their pragmatic abilities. In contrast, pragmatic accounts assume the ME response itself is based on pragmatic processes (Clark 2015; Diesendruck and Markson 2001). In this case, one would *not* assume participants to show a divergent pattern of performance on the ME task and on tasks assessing their pragmatic abilities. Instead, a population of children with advanced pragmatic skills should not face any difficulties with the ME task (as long as the testing conditions are adequate to assess their word learning strategies).

In the current study, we thus tested comparable samples of mono‐ and bilingual 3‐year‐olds and adults (as a validation group) with the same first language (German) and similar living surroundings, in comfortable test contexts (in their homes, with one parent being fluent in the test language). We assessed their disambiguation and retention performance in two conditions (within‐subjects): In the ME condition, a novel label was used in the presence of one novel and one familiar object. In the pragmatic condition, we presented two novel objects and the referent of the novel word could be pragmatically inferred based on common ground information (discourse novelty; see, e.g., Bleijlevens et al. 2023; Bohn et al. 2022).

With our design, we aimed to address how robust and persistent mono‐ and bilingual differences are in preschoolers, and how specific they are to the proposed areas. We predicted the following: First, if differences between language groups are robust and specific to the ME context because bilinguals learn that ME is not a reliable word learning strategy for them (Kalashnikova et al. 2018), then we expected bilingual 3‐year‐olds to be less likely to show the ME effect and subsequent retention than their monolingual peers. Second, if bilingual advantages are robust and extend to children's use of common ground information, bilingual children should outperform monolinguals in the pragmatic condition—potentially regarding both, referent disambiguation and retention. However, if differences between mono‐ and bilingual children are due to other factors, such as the test language and sampling biases, rather than the application of different word learning strategies, we would not expect any differences between both groups in our study with highly comparable mono‐ and bilingual samples.

## 2 Method

We preregistered the experimental design, procedure, sample sizes, and statistical analyses on OSF (https://osf.io/9epuw/). The complete study materials, data, analysis scripts, and details regarding the sample size calculation, the counterbalancing/randomization plan, and results are accessible on OSF as well. This project has been approved by the ethics committee of the Institute for Psychology, University of Göttingen (project number 317b).

### 2.1 Participants

#### 2.1.1 Children

The final sample for the main analyses included 74 typically developing 3‐year‐old children (36–47 months, *M* = 41.0, SD = 3.6; 31 females, 42 males, 1 without gender indication): 37 monolingual and 37 bilingual children. Eight additional children participated but were not included in the main analyses because they did not meet the bilingualism criteria (for details see Appendix D).

In addition, 10 children participated, but were excluded based on our pre‐registered exclusion criteria: technical issues (1), at least one mistake on familiarization trials (3) and uncooperative behavior (5) or because they did not provide any data in test trials (1).

The final samples of mono‐ and bilingual children were highly comparable. All children, both mono‐ and bilinguals, (a) had German as their first language (or as one of their first languages, in cases when parents stated that their child's input was more or less balanced across languages), (b) were recruited from the same data bases and living in similar surroundings in German university cities, and (c) had at least one parent who was fluent in German and present during the test session.

##### 2.1.1.1 Criteria for Mono‐ and Bilingualism

In a short, structured interview, we asked parents to indicate (a) the first language of their child, (b) whether they would describe their child as being raised bilingual, (c) the additional languages (beyond German) of their child, and (d) the estimated percentage of time their child is exposed to each language in their daily life.

Children were included in the bilingual group if they were exposed to at least one additional language regularly by one of their parents, constituting at least 20% of their language input. Additional languages included English, Spanish, Russian, Chinese, Danish, French, Polish, Turkish, Japanese, Dutch, Italian, Arabic, Romanian, Portuguese, Czech, and Bulgarian. The included bilingual children were exposed to an additional language on average for 42.9% of the time (SD = 13.6, range = 20%–70%).^3^ Children included in the monolingual group were raised by German‐speaking parents and had no regular contact with any other languages.

##### 2.1.1.2 Recruitment

Families were recruited from databases of German‐speaking children whose parents previously had expressed interest in child development studies. The participating children lived in German university cities and their surroundings. Further demographic data were not collected due to the local data protection rules (see Singh et al. 2024).

We determined the sample size of 74 children a priori via data simulation. The goal was to obtain 0.8 power to detect the assumed effect size for the comparison of mono‐ and bilinguals’ retention performance in the ME condition. The estimations for some of the parameters needed relied on data from another study (Bleijlevens et al. 2023). For details see https://osf.io/6f527.

#### 2.1.2 Adults

The final sample included 86 adults with German as their first language (19–72 years, *M* = 36.2, SD = 12.7 years; 36 females, 50 males; 77 White, 4 Asian, 3 Mixed, 1 Black, 1 Other): 43 monolinguals and 43 bilinguals. We determined this sample size a priori via data simulation. We aimed to obtain 0.8 power to detect the assumed effect size for the comparison of mono‐ and bilingual adults’ retention performance in the Pragmatic condition. The estimations for some parameters relied on data from another study (Bleijlevens et al. 2023). For details see https://osf.io/vx7b2. Adults were recruited via an online platform (www.prolific.com) and compensated for their participation at the recommended rate (£9/h).

##### 2.1.2.1 Criteria for Mono‐ and Bilingualism

Bilingual adults indicated on Prolific that their first language was German, that they were raised with two or more languages and are fluent in their native language (German) as well as at least one other language. Additional languages for bilinguals included Slovakian, English, Polish, French, Italian, Turkish, Bengali, Spanish, Vietnamese, Lebanese, Czech, Arabic, Danish, Dutch, Japanese, Russian, Swedish, and Urdu. See Appendix A for further information regarding adults’ language backgrounds, including the age of acquisition, and the percentage of current average exposure, usage and proficiency in each language.

Monolinguals indicated that their first language was German and that they were raised with their native language only. The monolingual adults included in the final sample also indicated that their country of birth was Germany, that they were still living in this country, and that they were exposed to the German language at least 90% of the time. Additional languages for these monolinguals were English, French, Spanish, Persian, and Latin.^4^ For further information on the recruitment of monolingual adults and on why the inclusion criteria used in the final sample of monolingual adults differ from the pre‐registered ones see Appendix D, which also reports the analyses of the adult samples based on the pre‐registered inclusion criteria.

### 2.2 Design

We used a 2 (condition: ME vs. pragmatic) × 2 (monolingual vs. bilingual) factorial design with conditions being tested within‐subjects. Children were tested in one test trial per condition and adults in two.

### 2.3 Stimuli

For auditory stimuli, we recorded three female German native speakers, one for each animal speaker in the experimental phases. Two non‐words (“ergi” and “sude”), that matched German phonology, served as novel labels in the referent disambiguation trials and six German known words served as labels in practice (“apple,” “house,” “flower,” and “bus”) and familiar‐label trials (“ball,” and “shoe”). The familiar distractor for the ME task was a car (and for adults additionally a flower). Based on the Wordbank (M. C. Frank et al. 2017), each of these words (those used as labels in practice and familiar‐label trials, as well as the word car) is, on average, produced by 94%–100% of 2.5‐year‐old German‐speaking children and expected to be well known to all of our 3‐year‐olds.

For visual stimuli, we used unknown object images from the NOUN database (Horst and Hout 2016), images provided by Bohn et al. 2022; see also Bleijlevens et al. 2023) and open source material. Videos were created via Powerpoint.

### 2.4 Procedure

**FIGURE 1:**
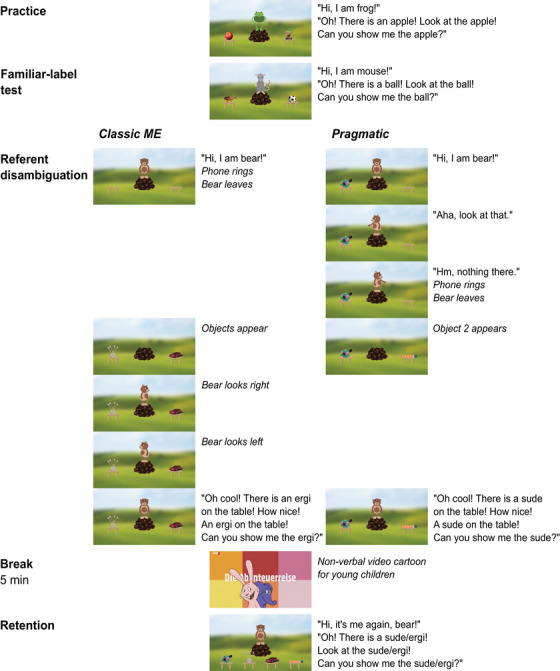
Experimental procedure: example trials for each phase in both conditions. The wording of the test question was constant across conditions, as were the main elements involved in the overall scene (e.g., bear leaving in response to a phone ringing, bear turning toward each table before asking the test question etc.).

The study was conducted online (due to the Covid‐19 pandemic). The child version was a synchronous online study (using BigBlueButton video conferencing), the adult version was an asynchronous online study presented via Labvanced (Finger et al. 2017). Participants watched short, animated videos which asked them to point to different objects. We video‐recorded children's, but not adults’, testing sessions. After providing informed consent, each participant was presented with 10 (children) or 12 (adults) trials in four experimental phases: Practice (children: 4 trials, adults: 2 trials), Familiar‐label test (2 trials), Referent disambiguation (children: 2 trials, adults: 4 trials) and Retention (children 2 trials, adults: 4 trials; Figure 1). We created 16 experimental versions for counterbalancing/randomization of the factors in the tasks (for details see the descriptions of each task type below).

On each trial, an animal speaker, located at center stage and looking straight ahead, asked for one of the presented objects. Adult participants directly clicked on the objects on the screen. Children selected objects by pointing at them. A letter then appeared underneath each of the objects on the screen and parents were instructed to indicate their children's choice by reading aloud the letter displayed under the chosen object. Parents and the experimenter were not permitted to help or interfere in children's choices in any way (except in Practice trials). When children did not respond, the experimenter encouraged the participant by asking “Just choose what you think is right.” After the main experiment, children participated in a German receptive vocabulary test and adults were asked to describe the selection strategies.

#### 2.4.1 Practice

Frog introduced herself. Then, on each trial, two known objects appeared on top of the screen and descended until they rested each on one of the two empty tables in front of frog. Frog asked the participant to point at a specific object by asking “Oh, there is a [known label]! Look at the [known label]. Can you show me the [known label]?” The experimenter interacted with the child until she picked the correct object and choices were followed by positive feedback from frog.

Practice trials were presented in fixed order (children: apple‐house‐flower‐bus; adults: bus‐house), and target locations (children: left‐right‐right‐left; adults: left‐right). Target locations were, thus, fully counterbalanced for each participant.

#### 2.4.2 Familiar‐Label Test

Mouse introduced herself. In each of the two familiar‐label trials, two known objects fell down on the two empty tables in front of her, followed by mouse's request to show one of them to her. In contrast to the practice trials, participants did not receive any feedback. Subjects who made at least one mistake in familiar‐label trials were excluded from analyses.

The object pairings (ball & duck, shoe & banana) and targets (ball, shoe) were fixed. The order of trials and target locations were randomized, either across experimental versions (for children) or in‐the‐moment by the experimental platform (for adults). Within participants, target locations were fully counterbalanced, with one target being presented on the left table and one on the right.

#### 2.4.3 Referent Disambiguation

Bear introduced herself. The following procedure within each trial depended on the condition. Children were presented with one trial per condition in counterbalanced order, adults with two.

##### 2.4.3.1 ME Condition

A phone started ringing and bear left the scene, disappearing inside the hill. Then, two objects, one familiar and one unfamiliar object, appeared on top of the screen and descended until they rested on the tables. Bear reappeared and turned toward each table in turn. Then, bear looked straight ahead and said excitedly “Oh cool! There is a [novel label] on the table! How nice, a [novel label] on the table! Can you show me the [novel label]?”.

##### 2.4.3.2 Pragmatic Condition

One object (the distractor) was laying on one of the two tables. Bear turned to each table. While looking and pointing at the empty table she said “Hm, nothing there”, and while turning to the occupied table she said “Aha, look at that”. A phone started ringing, and bear left the scene by disappearing inside the small hill. Meanwhile, a second novel object fell down and rested on the empty table, and then bear reappeared. Just as in the ME condition, bear then looked straight ahead and said excitedly “Oh cool! There is a [novel label] on the table! How nice, a [novel label] on the table! Can you show me the [novel label]?”

In the child study, across experimental versions, we counterbalanced the order of conditions, labels, and gaze directions (first to target/distractor side), and we randomized target locations (left/right) and the assignment of unknown objects to a role in the experiment (target or distractor in the Pragmatic task, or distractor in the ME task). In the adult study, which included 4 instead of 2 referent disambiguation trials, all of these variables were randomized across versions. We fully counterbalanced target locations within participants, and gaze directions within participants and conditions.

#### 2.4.4 Break

A children's non‐verbal video cartoon was played, serving as a time delay of 5 min prior to retention trials (see, e.g., Horst and Samuelson 2008).

#### 2.4.5 Retention

Participants were presented with one retention trial per newly learned label (i.e., two retention trials for children and four for adults). In each trial, bear was standing behind four tables when four objects fell down onto them. Without changing her frontal gaze direction, bear said “Oh, there is a [novel label]! Look at the [novel label]! Can you show me the [novel label]?”.

The four presented objects were identical in both conditions. In the child version, we presented all four objects they had encountered in the two referent disambiguation trials: both target objects and both distractor objects from the “ergi” and “sude” disambiguation trials. Across the experimental versions for children, we counterbalanced the label order (ergi/sude first), the correspondence of label order relative to the label order in referent disambiguation trials (same/different), and object locations, such that across these versions, each object was presented equally often at each position and changed its position between trials. In the adult version, we extended this approach to the four presented trials. For details see Appendix B.

#### 2.4.6 Receptive L1 Vocabulary Test (Children Only)

Following the main experiment, children participated in a German receptive vocabulary test for 3‐ to 8‐year‐olds (Bohn et al. 2023), including 20 trials.^5^ On each trial, four different pictures were presented on screen and a voice asked the child to point at one of them. Note that only part of our sample (20 monolinguals, 19 bilinguals) provided data on this test, because we only started implementing the test later in the data collection process and because some children failed to concentrate after the main study.

#### 2.4.7 Selection Strategies (Adults Only)

At the end of the experiment, we asked adults to describe how they decided on the object they selected in each condition (one question per condition). The question was accompanied by a screenshot of the participant's first referent disambiguation trial in that condition (in counterbalanced order). We asked, for example, “Please think back to the first game with bear: Could you shortly describe, how you decided which object ‘ergi’ is referring to in the situation on the picture?” and participants answered in an open text field.

### 2.5 Measures

#### 2.5.1 Correct Choices in Disambiguation Trials

We measured object choices for adults via their mouse clicks, and for children via their pointing gestures, confirmed by parents’ reading out of the corresponding letter. For referent disambiguation trials, we coded *correct choices*, for example, selecting the novel object (1) instead of the known/pre‐exposed one (0).

#### 2.5.2 Consistent Choices in Retention Trials

For retention trials, we coded *consistent choices*, that is, whether (1) or not (0) participants selected the same object they had previously selected in the corresponding disambiguation trial amongst four different objects (the target and distractor object from the previous corresponding disambiguation trial, and the target and distractor object from a previous disambiguation trial in the other condition).

#### 2.5.3 Adults’ Response Times

We measured adults’ response times in referent disambiguation and retention trials. Response times started with the first label onset and ended with their mouse click on one object.

#### 2.5.4 Adults’ Selection Strategies

A blinded coder assigned adults’ descriptions of their selection strategies to one of five pre‐registered categories: “speaker intent,” “nameability,” “familiarity,” “perceptual features,” and “explicit guessing” (Table 1). Reliability coding by a second blinded coder for all trials revealed 82% agreement. Cases of disagreement (31 out of 172) were discussed with a third coder until a joint decision was reached. The majority of disagreements were due to either assignments to closely related categories (i.e., disagreement between nameability and familiarity (*n* = 6) or between perceptual features and explicit guessing (*n* = 4)), or due to one coder, but not the other, refraining from assigning any category (*n* = 15).

**TABLE 1:** Categories of adults’ specific reasoning strategies.

Strategy	Explanation	Examples
Speaker intent	Reasoning based on the speaker, her behavior/intentions	“One object was already present and bear has seen it, but not named it. Then bear was surprised when the second object appeared—therefore it should be this one.”
Nameability	Reasoning based on the nameability of an object	“The other object already has a name.”
Familiarity	Reasoning based on the participant's familiarity with the object	“I am clearly familiar with one of the objects, therefore it must have been the one I didn't know.”
Perceptual features	Reasoning based on objects’ perceptual (visual/auditory) properties or salience	“The word seemed to fit the shape of the individual elements of the object.”
Explicit guessing	Indication of own ignorance/ selection based on intuition	“purely intuitively”/ “I don't know the word toma, so I just guessed”

*Note*: As preregistered, we distinguished the categories “nameability” and “familiarity.” However, we realized that many given answers were in line with both categories (e.g., “It can't be the car”), because they do not differentiate if the distractor was excluded based on its name or familiarity. In these cases, we decided to code “nameability” whenever the object's name was mentioned, leading to a high number of “nameability” and a relatively low number of “familiarity” codings.

### 2.6 Statistical Analysis

For data analysis, we used R (version 4.2.1; version 2023.6.0.421). Appendix C lists all functions and packages used. The data set, R scripts, analysis results and assumption tests are accessible on OSF (https://osf.io/9epuw). If not stated otherwise, we followed our preregistered analysis plan and the model assumptions were met.

Before interpreting model parameters, we tested for the overall effect of our fixed effects for each model with more than one predictor by using Likelihood Ratio Tests comparing the fit of the full model to that of a null model, lacking the predictors of interest. This way, we avoided “cryptic multiple testing” (Forstmeier and Schielzeth 2011).

As preregistered, we removed non‐significant interactions from full models in a stepwise fashion, starting with non‐significant higher‐order (3‐way) interactions (e.g., language background × condition × age group), and followed by non‐significant lower‐order (2‐way) interactions (e.g., language background × condition).

#### 2.6.1 Correct Object Choices in Referent Disambiguation

**FIGURE D1:**
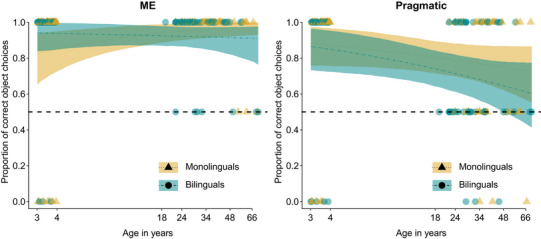
Correct object choices in referent disambiguation trials by (continuous) age. Gray triangles (monolinguals) and dots (bilinguals) show the proportions of correct object choices per participant, based on trials in which any selection was made (children: *n*_monolingual_ = 73, *n*_bilingual_ = 70; adults: *n*_monolingual_ = 172, *n*_bilingual_ = 172). Dotted (monolinguals) and dash‐dotted (bilinguals) lines represent the fitted values; and darker (monolinguals) and lighter (bilinguals) polygons show the 95% confidence intervals, both calculated via bootstrapping with 1000 boots. The values base on the GLMM with binomial error distribution predicting correct choices in disambiguation trials by language background (monolingual/bilingual), condition (ME/Pragmatic), age (continuous, log‐ and z‐transformed), and all of their possible interactions as predictors, the speaker's gaze order (first to target/distractor) as a control variable and random intercepts for participants.

To test whether participants’ performance in referent disambiguation trials differed between language backgrounds or conditions, we fitted a GLMM with binomial error distribution. We predicted participants’ correct object choices by language background (monolingual/bilingual), condition (ME/Pragmatic), age group (children/adults), and all of their possible interactions. Additionally, we added the speaker's gaze order (first to target/distractor) as a control variable and random intercepts for participants. This analysis differed from the preregistered one in that we replaced the predictor continuous age (in years) by age group. The new model eased the interpretation and description of the results, but revealed the same pattern of results as the preregistered one (see Appendix D: Figure D1 and Table D1).

#### 2.6.2 Adults’ Response Times

To test for effects of language background and condition on adults’ processing speed, we fitted a LMM. We predicted adults’ log‐transformed response times by language background, condition and their interaction. We added age (z‐transformed)^6^ and the speaker's gaze order as control variables, and random intercepts for participants.

#### 2.6.3 Adults’ Selection Strategies

To test adults’ differential use of strategies across conditions, we fitted a multinomial mixed effects model. We predicted adults’ strategies by language background, condition, and their interaction, and added random intercepts for participants.

#### 2.6.4 Consistent Object Choices in Retention

**FIGURE D2:**
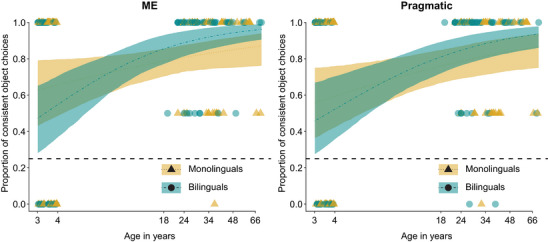
Consistent object choices in retention trials by (continuous) age. Gray triangles (monolinguals) and dots (bilinguals) show the proportions of consistent object choices in retention trials per participant, based on trials in which any selection was made (children: *n*_monolingual_ = 73, *n*_bilingual_ = 70; adults: *n*_monolingual_ = 172, *n*_bilingual_ = 172). Dotted (monolinguals) and dash‐dotted (bilinguals) lines represent the fitted values; and darker (monolinguals) and lighter (bilinguals) polygons show the 95% confidence intervals, both calculated via bootstrapping with 1000 boots. The values base on the GLMM with binomial error distribution predicting consistent choices in retention trials by language background (monolingual/bilingual), condition, age (continuous, log‐ and z‐transformed), and all of their possible interactions as predictors, and random intercepts for participants.

To test whether participants’ retention performance was affected by language group and/or conditions, we fitted a GLMM with binomial error distribution. We predicted participants’ consistent object choices by language group, condition, age group, and all of their possible interactions. We added random intercepts for participants. As above, we decided to ease the interpretation and communication of the results by replacing the preregistered predictor continuous age (in years) by age group. This model revealed the same pattern of results as the preregistered one (see Appendix D: Figure D2 and Table D2).

#### 2.6.5 Exploratory Analysis

First, we tested whether mono‐ and bilinguals differed in their L1 (German) vocabulary size. We fitted a GLMM with binomial error distribution, predicted their correct choices in the vocabulary test (on a trial basis) by language background, and added random intercepts for participants.

Second, to assess whether different measures of language background/ bilingualism (see Appendix A) may have affected adults’ disambiguation and retention, we exploratorily ran the main analyses described above (object choices in disambiguation and retention trials, and response times in disambiguation trials) again and replaced the main bilingualism predictor (raised bilingual) with all of our alternative measures. The other bilingualism measures revealed a similar pattern of results, with two exceptions: With increasing L1 exposure and usage (compared to their exposure to/ usage of their additional languages), adults’ performance in the ME disambiguation task significantly increased (see Supporting Information: Supplement A for details).^7^

## 3 Results

### 3.1 Correct Object Choices in Disambiguation Trials

**FIGURE 2:**
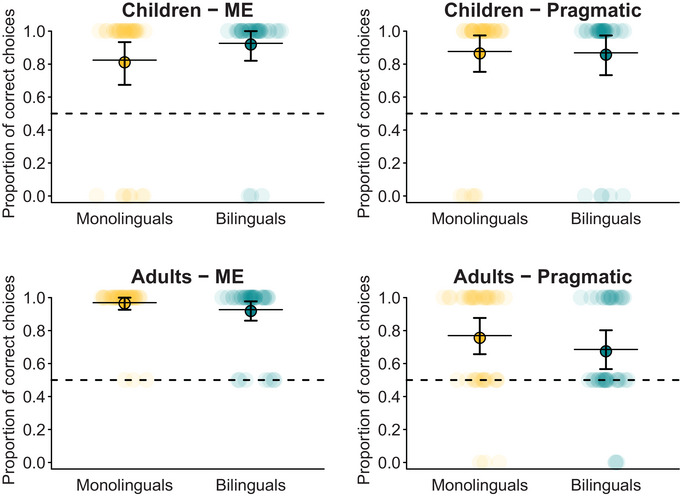
Correct object choices in referent disambiguation trials. Transparent dots represent the proportions of correct object choices per participant, based on those trials in which any choice was made (children: *n*_monolingual_ = 74, *n*_bilingual_ = 72; adults: *n*_monolingual_ = 172, *n*_bilingual_ = 172) and circled dots the aggregated proportions per group and condition. Horizontal lines indicate the predicted probabilities; and vertical lines the 95% confidence intervals, both obtained by the GLMM and calculated via bootstrapping with 1000 boots.

The binomial GLMM on participants’ correct object choices in disambiguation trials (correct choice ∼ language background * condition * age group + gaze order + (1|participant)) revealed that both children and adults selected the target object significantly above chance level in both conditions (Figure 2: the bootstrapped confidence intervals do not include chance level).

As preregistered, in a stepwise fashion, we first removed the non‐significant 3‐way interaction (language background × condition × age group: *b* = 1.54, SE = 1.29, *p* = 0.232), and then the non‐significant 2‐way interactions (language background × condition: *b* = −0.09, SE = 0.60, *p* = 0.878; language background x age group: *b* = −0.94, SE = 0.64, *p* = 0.141). See Table 2 for the results of the full model. The final reduced model included all main effects and only the significant interaction of condition and age group (*b* = −1.91, SE = 0.62, *p* = 0.002).

**TABLE 2:** Model predicting correct choices in referent disambiguation trials by language background, condition, age group, their interactions, and gaze order.

	Estimate	SE	*p*	95% CI
Reference groups: monolinguals, ME condition, children				
Intercept	1.69	0.48	<0.001	0.86, 2.80
Language background	0.99	0.76	0.192	−0.40, 10.26
Condition	0.42	0.65	0.522	−0.88, 2.23
Age group	1.91	0.74	0.010	0.59, 11.06
Gaze order	−0.27	0.27	0.305	−0.82, 0.25
Language background × condition	−1.06	1.02	0.297	−10.29, 1.02
Language background × age group	−1.90	1.05	0.070	−12.06, 0.00
Condition × age group	−2.67	0.92	0.004	−11.75, −1.01
Language background × condition × age group	1.54	1.29	0.232	−1.07, 12.01
Reference groups: bilinguals, Pragmatic condition, adults				
Intercept	0.92	0.30	0.002	0.39, 1.56
Language background	0.43	0.37	0.250	−0.30, 1.21
Condition	1.77	0.47	<0.001	0.97, 3.03
Age group	1.11	0.56	0.049	0.12, 2.70
Gaze order	−0.27	0.27	0.305	−0.82, 0.25
Language background × condition	0.48	0.79	0.543	−1.21, 10.85
Language background × age group	−0.35	0.80	0.658	−2.17, 1.42
Condition × age group	−1.12	0.91	0.218	−3.19, 9.16
Language background × condition × age group	−1.54	1.29	0.232	−13.18, 1.08

*Note*: GLMM with binomial error distribution on participants’ correct choices in referent disambiguation trials with language background (monolingual/bilingual), condition (ME/Pragmatic), age group (children/adults) and all of their interactions as predictors, gaze order (first to target/distractor) as control variable and random intercepts for participants (SD = 0.54). *N*_observations_ = 490. *N*_groups_ = 160. Confidence intervals were obtained via bootstrapping with 1000 boots.

Overall, language background did not affect the performance in disambiguation trials (*b* = −0.24, SE = 0.28, *p* = 0.396). Children's performance did not differ between conditions (*b* = −0.03, SE = 0.49, *p* = 0.950), but adults performed significantly better in the ME than in the Pragmatic condition (*b* = −1.94, SE = 0.38, *p* < 0.001). In fact, adults performed significantly better than children in the ME condition (*b* = 0.97, SE = 0.49, *p* = 0.048), but significantly worse than children in the Pragmatic condition (*b* = −0.95, SE = 0.40, *p* = 0.019). The speaker's gaze order (i.e., which object the bear looked to first before requesting the target) did not affect performance (*b* = −0.28, SE = 0.27, *p* = 0.287). The reduced model described the data significantly better than the corresponding null model (*χ* ^2^(4) = 36.00, *p* < 0.001) and did not differ significantly from the full model including all interactions (*χ* ^2^(3) = 3.99, *p* = 0.262).

### 3.2 Adults’ Response Times

The LMM on adults’ response times (log.RT ∼ language background * condition + z‐age + gaze order + (1|participant)) did not reveal a significant interaction between language background and condition (*b* = 0.02, SE = 0.08, *p* = 0.782). Therefore, we interpreted the reduced model lacking this interaction. There was no significant effect of language background on adults’ response times (*b* = 0.08, SE = 0.10, *p* = 0.415). However, in line with their object selections, adults responded significantly faster in the ME (*M* = 7.3 s, SD = 3.7 s) than the Pragmatic condition (*M* = 8.5 c, SD = 4.1 s; *b* = 0.21, SE = 0.04, *p* < 0.001). There were no significant effects of adults’ age (*b* = 0.09, SE = 0.05, *p* = 0.085) nor the speaker's gaze order (*b* = −0.01, SE = 0.04, *p* = 0.827) on their response times. The reduced model described the data significantly better than the corresponding null model (*χ* ^2^(2) = 24.97, *p* < 0.001) and did not differ significantly from the full model including the interaction (*χ* ^2^(1) = 0.08, *p* = 0.782).

### 3.3 Adults’ Selection Strategies

**FIGURE 3:**
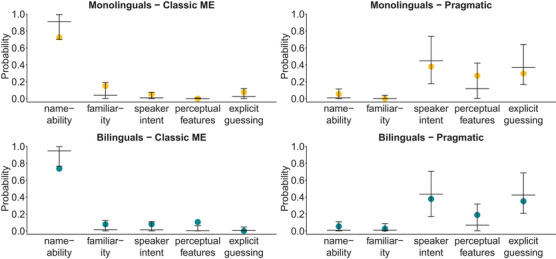
Adults’ reasoning strategies for referent disambiguation. Dots show the proportions of actual selection strategies for those trials in which adults indicated a strategy matching any of the five preregistered categories (monolinguals: *n*_ME_ = 40, *n*_Pragmatic_ = 37; bilinguals: *n*_ME_ = 38, *n*_Pragmatic_ = 37; 20 of the 172 trials included answers that did not match one of our categories). Horizontal lines indicate the predicted probability of this strategy by the multinomial mixed model and vertical lines their 95% confidence intervals.

The multinomial GLMM on adults’ selection strategies (strategy ∼ language background * condition + (1|participant)) revealed that mono‐ and bilinguals did not differ in the described strategies they used to disambiguate words in our tasks (Figure 3: confidence intervals of one language group include fitted values of the other). In the ME condition, both mono‐ and bilingual adults described strategies in line with the nameability category significantly more often than any other category. In the Pragmatic condition, strategies based on the speaker's intentions were the most prevalent of the five categories (described in 38% of the Pragmatic trials) for both mono‐ and bilinguals (Table 3). These “speaker intent” strategies were significantly more frequent than strategies based on the objects’ nameability, familiarity, or perceptual features. However, the number of adults who decided on an object by guessing and/or based on its perceptual features was still unexpectedly high.

**TABLE 3:** Adults’ reasoning strategies for referent disambiguation.

Strategy	ME		Pragmatic	
	Monolinguals	Bilinguals	Monolinguals	Bilinguals
Speaker intent	2 (5.0%)	3 (7.9%)	14 (37.8%)	14 (37.8%)
Nameability	29 (72.5%)	28 (73.7%)	2 (5.4%)	2 (5.4%)
Familiarity	6 (15.0%)	3 (7.9%)	—	1 (2.7%)
Perceptual features	—	4 (10.5%)	10 (27.0%)	7 (18.9%)
Explicit guessing	3 (7.5%)	—	11 (29.7%)	13 (35.1%)

*Note*: Percentage of coded strategies per language group and condition for those responses that were codable (*n* = 152 out of 172).

### 3.4 Consistent Object Choices in Retention Trials

**FIGURE 4:**
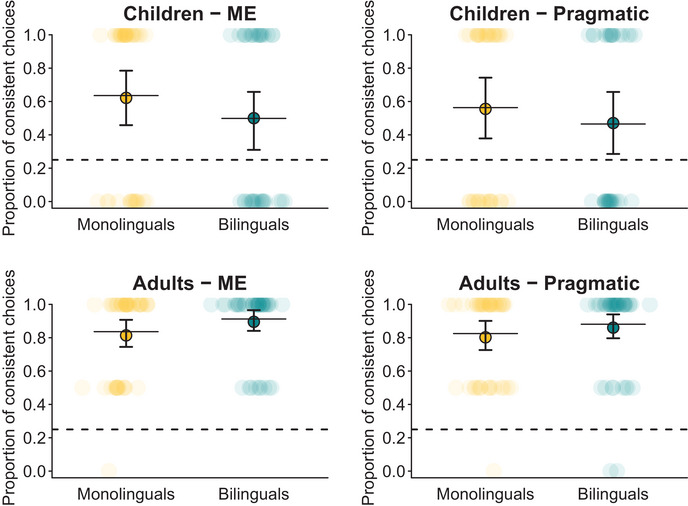
Consistent object choices in retention trials. Transparent dots represent the proportions of consistent object choices per participant, based on those trials in which any choice was made (children: *n*_monolingual_ = 73, *n*_bilingual_ = 70; adults: *n*_monolingual_ = 172, *n*_bilingual_ = 172) and circled dots the aggregated proportions per group and condition. Horizontal lines indicate the predicted probabilities; and vertical lines the 95% confidence intervals, both obtained by the GLMM and calculated via bootstrapping with 1000 boots.

The GLMM on participants’ consistent object choices in retention trials (consistent choice ∼ language group * condition * age group + (1|participant)) revealed that children and adults, both mono‐ and bilinguals, made consistent choices in retention trials above chance (i.e., 25%, due to the selection amongst four objects) in both conditions, as confirmed by the bootstrapped confidence intervals (Figure 4: confidence intervals do not include the chance level of 0.25).

The full model did not reveal a significant 3‐way interaction of condition × language background × age group (*b* = −0.43, SE = 0.95, *p* = 0.651). As preregistered, we first removed this interaction from the model, followed by the nonsignificant 2‐way‐interactions (condition × language background: *b* = −0.08, SE = 0.47, *p* = 0.870; condition × age group: *b* = 0.02, SE = 0.47, *p* = 0.961). See Table 4 for the results of the full model. The resulting reduced model included all main effects and only the significant interaction of language background × age group (*b* = 1.05, SE = 0.53, *p* = 0.048).

**TABLE 4:** Model predicting consistent choices in retention trials by language background, condition, age group, and their interactions.

	Estimate	SE	*p*	95% CI
Reference groups: monolinguals, ME condition, children				
Intercept	0.56	0.38	0.142	−0.17, 1.29
Language background	−0.56	0.53	0.291	−1.63, 0.35
Condition	−0.30	0.51	0.551	−1.33, 0.66
Age group	1.08	0.49	0.028	0.14, 2.02
Language background × condition	0.17	0.72	0.816	−1.27, 1.60
Language background × age group	1.27	0.72	0.078	−0.06, 2.80
Condition × age group	0.22	0.65	0.732	−1.00, 1.51
Language background × condition × age group	−0.43	0.95	0.651	−2.43, 1.38
Reference groups: bilinguals, Pragmatic condition, adults				
Intercept	2.00	0.36	<0.001	1.36, 2.75
Language background	0.45	0.46	0.323	−1.40, 0.44
Condition	0.34	0.48	0.475	−0.64, 1.43
Age group	−2.14	0.53	<0.001	−3.24, −1.16
Language background × condition	−0.26	0.63	0.675	−1.67, 0.97
Language background × age group	0.84	0.71	0.232	−0.61, 2.20
Condition × age group	−0.21	0.70	0.765	−1.72, 1.25
Language background × condition × age group	0.43	0.95	0.651	−1.38, 2.43

*Note*: GLMM with binomial error distribution on participants’ consistent choices in retention trials with language background (monolingual/bilingual), condition, age group (children/adults) and all of their interactions as predictors, and random intercepts for participants (SD = 0.70). *N*_observations_ = 487. *N*_groups_ = 160. Confidence intervals were obtained via bootstrapping with 1000 boots.

The model revealed that, although adults had to remember four word‐object‐links and children only two, adults’ performance in retention trials was significantly better than children's, and as suggested by the interaction, this effect was even stronger for bilinguals (*b* = 2.24, SE = 0.41, *p* = < 0.001) than for monolinguals (*b* = 1.19, SE = 0.37, *p* = 0.001). However, there were no significant effects of language background, neither for children (*b* = −0.48, SE = 0.40, *p* = 0.227) nor for adults (*b* = 0.57, SE = 0.35, *p* = 0.104), and no effect of condition (*b* = −0.20, SE = 0.23, *p* = 0.389). The reduced model described the data significantly better than the corresponding null model (*χ* ^2^(4) = 44.8, *p* < 0.001) and did not differ significantly from the full model including all interactions (*χ* ^2^(3) = 0.24, *p* = 0.972).

### 3.5 Receptive L1 Vocabulary Size (Exploratory)

The GLMM on children's correct choices in the vocabulary test (correct choice ∼ language background + (1| participant)) revealed that the bilingual children had a significantly lower vocabulary size in their first language German (*M* = 7.1, SD = 2.8) than the monolinguals (*M* = 11.0, SD = 2.3; *b* = −0.81, SE = 0.18, *p* < 0.001).

## 4 Discussion

In the current study, we tested whether mono‐ and bilinguals differ in the strategies and socio‐pragmatic skills underlying their word learning. We found that mono‐ and bilingual children (and adults) were equally successful at disambiguating and retaining novel word meanings in a ME task as well as a pragmatic task that required taking common ground into account. In contrast to prior suggestions, in our comparable samples of children with different language backgrounds, mono‐ and bilinguals did not differ in the strategies and pragmatic abilities they relied on to learn the meanings of novel words.

### 4.1 (No) Differences in Referent Disambiguation and Retention Using Mutual Exclusivity

Past research suggested differences in how mono‐ and bilinguals use ME to disambiguate novel word meanings and subsequently retain these labels (Kalashnikova et al. 2018). Here, we tested mono‐ and bilingual 3‐year‐olds’ (and adults’) disambiguation and retention of novel words in the ME task. Both groups were successful at inferring that the novel label referred to the novel object and retained the labels after a short delay—without any differences between language groups. Furthermore, both groups of children performed just as successfully in the ME task as in the pragmatic task (a task that did not include any known labels or objects and in which the correct referent could be inferred pragmatically).

For adults, exploratory analyses suggested that the proportion of exposure to (and usage of) the test language predicted their success in the ME disambiguation task. This finding highlights how measuring bilingualism in a rather continuous manner can potentially provide more fine‐grained insights into the effects of language experience on cognitive tasks. However, note that these findings were revealed in a large set of exploratory analyses (in which all other effects of interest did not reach significance), and not mirrored in the analyses based on participants’ self‐reported affiliation to the mono‐ or bilingual group. Thus, future research should investigate more systematically whether measures of bilingualism that are not based on arbitrary cut‐offs, may robustly affect adults’ (or children's) word learning (see, e.g., Rocha‐Hidalgo and Barr 2023 for the impact of cut‐offs between mono‐ and bilinguals on the reported differences in the literature, and Gullifer and Titone 2020, for using language entropy as a measure capturing the social diversity of bilingual language background and use).

The lack of performance differences between the mono‐ and bilingual groups contradicts the idea that bilinguals’ language experience, particularly their experience with cross‐language synonyms (i.e., many‐to‐one mappings) results in the development of different word learning strategies: It has been proposed that bilingual children may learn around their second birthday that ME as a word learning strategy is not reliable for them (Kalashnikova et al. 2018). However, in that case, we would have expected (a) that the differences between mono‐ and bilinguals’ performance in the ME task are especially pronounced in preschoolers and (b) that bilinguals perform worse in the ME than the pragmatic disambiguation task, since the impact of bilinguals’ language experience should have been specific to tasks involving word‐known distractors. In contrast, neither children's language background nor the experimental conditions affected their disambiguation and retention performance: Mono‐ and bilinguals performed equally well in disambiguating and learning novel word meanings, both in the ME task and the pragmatic task.

The results are in line with the idea that mono‐ and bilingual children have the same word learning strategies available, but this may have been masked by various other factors in previous studies. First, bilinguals’ vocabulary in the test language may have led to a weaker ME effect in bilingual infants, while the 3‐year‐olds tested here already had sufficient vocabulary in the test language to use ME to a similar extent as their monolingual peers. Second, bilingual children may not always feel comfortable in the test language, causing general performance differences that are not due to their bilingualism per se. To avoid this confound, (a) we tested comparable samples of mono‐ and bilinguals who had the same first language (German), (b) children had at least one parent who was fluent in that language and also present during the test session, and (c) children were tested at home in their familiar environment. Third, methodological issues may have influenced the pattern of results in the literature, including small and unbalanced samples whose performance was not always directly compared, but separately tested against a chance value. This potential overestimation of differences was prohibited here by testing a balanced and bigger sample and running a preregistered analysis that included a direct test of the effect of bilingualism.

Importantly, the fact that we did not find bilingual children to perform any different from monolinguals in our study was probably not because bilinguals tested here were “not bilingual enough”: The bilingual sample received at least 20% input in their additional language(s) which was provided daily by one of their parents. Additionally, just like in previous studies, we found bilinguals to have a smaller vocabulary in their first (and test) language. Nevertheless, there were no differences between mono‐ and bilingual's disambiguation and retention performance.

In sum, our study does not reveal any differences between mono‐ and bilinguals in using ME for word disambiguation and learning. Our comparable samples of mono‐ and bilingual 3‐year‐olds inferred and retained novel word‐object mappings in a ME task to the same extent. Their language background ultimately did not influence the strategies available to learn novel word meanings.

### 4.2 (No) Differences in Referent Disambiguation and Retention Using Pragmatic Reasoning

Another line of past research suggested advantages of bilingual children in social‐pragmatic and communicative skills that may be beneficial for understanding referential intent (e.g., Siegal et al. 2009; Wermelinger et al. 2017; Yow and Markman 2015). Here, we tested participants in a pragmatic condition in which the correct referent could be inferred pragmatically by considering common ground (specifically, discourse novelty): Since one object was already given in the common ground, the speaker's later excitement while using a novel label indicated that this label rather referred to the novel object. The data revealed that mono‐ and bilinguals (both children and adults) were similarly successful at making this pragmatic inference and retaining the new word‐object mapping after a delay.

While there was no difference in performance between mono‐ and bilinguals, we observed an unexpected difference between the two age groups: While children performed very well in our pragmatic disambiguation task, a subgroup of the tested adults showed difficulties in interpreting the pragmatic context. Their reduced performance in the pragmatic disambiguation task, as well as their strategy descriptions, indicated that these adults did not consider the common ground information, but instead guessed and/or selected an object based on its perceptual features. In contrast to the children, adults participated in an asynchronous study with no video‐record. Thus, some adult participants included in the final sample may have been inattentive, thereby missing crucial elements of the pragmatic context (i.e., that the bear had already looked and commented on one of the objects)—especially as during these elements participants were just meant to watch and not required to respond. Note that in previous work, adults had shown much better performance in almost identical tasks (Bleijlevens et al. 2023; Bohn et al. 2022) except that here the crucial elements were presented twice^8^ thereby reducing the chance that participants might miss them due to inattention.

In neither age group, however, was there any evidence for a bilingual pragmatic advantage in using common ground to disambiguate or learn novel word meanings. How does this finding fit in the picture drawn by previous research?

There are different explanations for the current pattern of results. First, bilinguals may not actually possess any pragmatic advantages (see also Antoniou et al. 2020; van Wonderen et al. 2023). Contrary to some proposals in the literature, communicative challenges of bilinguals may not lead to improved pragmatic skills compared to monolinguals. If true, positive findings from other studies may present sampling and performance issues.

Second, bilinguals may possess pragmatic advantages that we failed to detect in our task due to ceiling effects (given that monolinguals already showed a quite high performance in our pragmatic disambiguation task). Future research should investigate the performance of mono‐ and bilingual children in a more demanding pragmatic word‐learning task that induces more variation in children's performance.

Third, bilingual pragmatic advantages may exist, but be more specific either to certain circumstances or to other areas of pragmatic skills. For example, the enhancement of pragmatic skills may only manifest in populations who are confronted with communicative challenges to a stronger extent. Given that in our sample, one parent was fluent in each of the child's languages, respectively, children may not have faced too challenging experiences that would result in the need to focus on different social cues to understand their communication partner. Alternatively, it is possible that the challenging communicative experiences of bilingual children train, for example, their ability to take their interlocutor's perspective or affect their weighing of social cues in ambiguous contexts (Fan et al. 2015; Liberman et al. 2017; Yow et al. 2017; Yow and Markman 2015). However, they may show no effect on the fundamental skills that are underlying every child's word learning (regardless of language background), such as the ability to consider common ground information during discourse (see e.g., Bleijlevens et al. 2023; Liebal et al. 2009; Matthews et al. 2006).

To conclude, children (and adults too) need socio‐pragmatic understanding in many areas of their lives, including the area of language acquisition. Irrespective of their language background, they can use these general pragmatic skills to understand the behavior and communication of others and to learn the words of our language(s). Our study revealed no advantage for bilingual children and adults in using common ground to identify the referents of novel words and retaining them. This seems plausible given that interpreting the pragmatic context is so crucial for children's social lives, regardless of whether they learn one or many languages. More research is needed to determine if bilingual pragmatic advantages do not exist at all or apply to specific pragmatic areas, for example, those in which understanding the referential intent requires correct weighing of different cues.

### 4.3 The Mechanisms Behind Children's Referent Disambiguation

There is a long‐standing theoretical debate focusing on how young children succeed in disambiguating novel word meanings in tasks such as the ME task. Three theoretical approaches have been put forward: While *lexical accounts* propose that children rely on lexical constraints such as the ME bias (assuming concepts to have only one name, e.g., “the car cannot have two names”; Golinkoff et al. 1994; Markman and Wachtel 1988), *socio‐pragmatic accounts* assume children to use their general socio‐cognitive abilities to interpret the speaker's intentions (e.g., “if she meant the car, she would have used the conventional word”; Clark 2015; Diesendruck and Markson 2001; Tomasello 2010), and *associative accounts* explain children's behavior by associative processes such as the attraction to novelty (Mather and Plunkett 2012; Smith 2000; but see Bleijlevens et al. 2023; Bleijlevens and Behne 2024). How can our findings from the ME task add to the debate about the mechanisms behind children's referent disambiguation?

Since bilingualism and experiential effects in general were mostly not explicated in the initial formulations of each theoretical proposal (see e.g., Markman et al. 2003), our data cannot provide clear evidence for or against certain theoretical approaches. However, some researchers made specific predictions about the role of linguistic experience that are not in line with our findings. One version of the lexical constraint accounts claims that children's development of the ME constraint is based on their experience with one‐to‐one mappings. According to this idea, bilingual children should not acquire the ME principle because they learn more than one word per concept (e.g., Byers‐Heinlein and Werker 2009; Houston‐Price et al. 2010), at least across languages. Similarly, associative network accounts could predict a poorer ME performance for cases in which concepts have more than one label, because the associative network is shaped by language experience and the structure of bilinguals’ lexicon is not sufficient to use ME (McMurray et al. 2012).

The illustrated predictions by both lexical and associative accounts are not in line with our findings. However, our data may be compatible with a specific version of these accounts in which bilinguals separate their languages and ME is only applied within a language. This is in line with studies showing that mono‐ and bilinguals use ME only within and not across languages (Au and Glusman 1990) and studies showing a weakened ME effect when the novel target word is presented in isolation versus embedded in a carrier phrase that provides additional information regarding the word's language affiliation (Rochanavibhata et al. 2022). This discussion ultimately leads to the question of how and when bilingual children separate their languages (see, e.g., Byers‐Heinlein 2014).

Finally, the pragmatic word learning accounts would predict to observe a ME effect whenever children can assume that the speaker knows the conventional word for the known (distractor) object and would use it if she wanted to refer to it (Clark 2015; Diesendruck 2005). Socio‐pragmatic accounts would therefore not predict any differences between mono‐ and bilingual children in our design in which the speaker was monolingual and thus knowledgeable regarding the distractor labels. The pragmatic account is thus the only one which is unconditionally supported by our data.

### 4.4 Conclusion

The current study investigated whether mono‐ and bilingual children's language experiences lead to differences regarding the strategies and pragmatic skills underlying their word learning. In contrast to prior suggestions, monolingual children were not more likely than their bilingual peers to disambiguate or retain novel words in a ME task. Bilingual children's language experience did not prevent them from developing or maintaining the ME strategy to learn novel words. Additionally, they were not more successful than monolinguals in using pragmatic common ground information to disambiguate and retain novel word‐object mappings. Bilinguals’ language experience and potential communicative challenges did not result in advanced pragmatic abilities that underly their word learning.

Our findings suggest that comparable samples of mono‐ and bilingual children seem to develop the same strategies and pragmatic abilities to disambiguate and learn the meanings of novel words: They can make use of their lexical knowledge and socio‐cognitive understanding to infer the meanings of novel words in their language—regardless of whether they learn only one or several languages.

## Conflicts of Interest

The authors declare no conflicts of interest.

## Ethics Statement

This research project has been approved by the ethics committee of the Institute for Psychology, University of Göttingen (project number 317b).

## Supporting information



Supporting Information

## Figures and Tables

**FIGURE D3:**
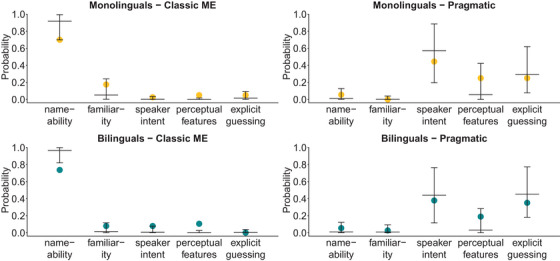
Adults’ reasoning strategies for referent disambiguation. Dots show the proportions of actual selection strategies for those trials in which adults indicated a strategy matching any of the five preregistered categories (monolinguals: *n*_ME_ = 40, *n*_Pragmatic_ = 36; bilinguals: *n*_ME_ = 38, *n*_Pragmatic_ = 37; 21 of the 172 trials included answers that did not match one of our categories). Horizontal lines indicate the predicted probability of this strategy by the multinomial mixed model and vertical lines their 95% confidence intervals.

## Data Availability

This study was preregistered on OSF; see https://osf.io/x9aej. The complete data, analysis code and experimental stimuli are accessible there as well (https://osf.io/9epuw/).

## Endnotes

1 See also Luk (2023), for a nuanced discussion on the problematic overgeneralizations and value judgments that framing research on the potential impact of different linguistic experiences in terms of bilingual (dis‐)advantages can bring about.

2 In line with this, some research showed that it was specifically those bilinguals who knew many translation equivalents that did not show the ME bias (Byers‐Heinlein and Werker 2013). However, the empirical evidence is sparse, and other studies failed to find the suggested relationship (e.g., Frank and Poulin‐Dubois 2002; Houston‐Price et al. 2010; Weatherhead et al. 2021).

3 Note that these values are based on the responses of 19 (out of 37) parents who indicated concrete estimations for their children's language exposure.

4 Note that in Germany it is very common to learn and be exposed to additional languages (especially English) from early on. This is also reflected by monolinguals’ indicated second languages: These mirror the requirements of the German school system. While the language of schooling is German, students learn English as a mandatory second language, and often a third language (predominantly French, Spanish, or Latin), too.

5 Note that this was an earlier version of the test including only 20 trials (instead of 22 in the final version).

6 We preregistered to log‐transform age for this analysis. However, while this makes sense in a model including both children's and adults’ data, it is not necessary in a model including only adults’ data. Note that this decision did not change the results.

7 Note, however, that in this set of exploratory analyses, we ran 12 statistical models and inspected 24 effects of interest, of which 2 reached significance.

8 To achieve maximal comparability to the ME condition, we reduced the length of the pragmatic condition compared to the scene used in Bohn et al. (2022) and Bleijlevens et al. (2023). While in those studies, the speaker left and entered the scene twice to comment on both the empty table and the “old” object in the scene, she did so only once in our task. Thus, in our task the contrast in the speaker's excitement between the old and the new object was less pronounced, potentially leading to a more subtle pragmatic context. The procedural changes inadvertently resulted in a task more susceptible to be negatively affected by brief moments of participant inattention. In previous work using the longer original scenes adults’ participant showed much better performance regarding both in their referent disambiguation and in their explanation of the strategies used.

## Appendix A Continuous Measures of Bilingualism in Adults

We asked adult participants to indicate further specifics of their language(s). For each of their languages, we asked them to indicate the following variables: the kind of language, age of acquisition, the percentage of current and average exposure time, the percentage of current and average usage time, and their proficiency in each language (on a 7‐point Likert scale: “very low”—“low”—“adequate”—“moderate”—“good”—“very good”—“excellent”). Table A1 shows the descriptive data for all of these additional measures in the final adult sample.

**TABLE A1:** Adults’ language specifics.

	Languages				
	L1	L2	L3	L4	L5
Monolinguals	German (43)	English (34)	French (3) Latin (1) Persian (1) Spanish (1)	French (2)	—
Bilinguals	German (42) Slovakian (1)^1^	English (28) Polish (3) French (2) Italian (2) Turkish (2) Bengali (1) German (1) Lebanese (1) Spanish (1) Vietnamese (1)	English (11) French (3) Spanish (3) Czech (2) Arabic (1) Danish (1) Dutch (1) Japanese (1) Polish (1) Russian (1) Swedish (1) Turkish (1) Urdu (1)	French (3) English (2) Latin (2) Dutch (1)	Low German (1)

	Age of acquisition (years)				
	L1	L2	L3	L4	L5
Monolinguals	Range = 0–5 *M* = 0.7 SD = 1.1	Range = 1–16 *M* = 9.0 SD = 2.7	Range = 12–34 *M* = 20.2 SD = 10.4	Range = 7–15 *M* = 11.0 SD = 5.7	—
Bilinguals	Range = 0–5 *M* = 1.2 SD = 1.4	Range = 0–18 *M* = 6.9 SD = 4.8	Range = 0–27 *M* = 9.8 SD = 7.2	Range = 3–15 *M* = 10 SD = 3.6	Range = 0–0 *M* = 0 SD = 0

	% Exposure^2^				
	L1	L2	L3	L4	L5
Monolinguals	Range = 90–100 *M* = 95.3 SD = 4.0	Range = 0–10 *M* = 6.2 SD = 3.4	Range = 0–1 *M* = 0.5 SD = 0.6	*—*	*—*
Bilinguals	Range = 4–100 *M* = 57.0 SD = 24.4	Range = 5–90 *M* = 32.1 SD = 19.8	Range = 0–55 *M* = 15.1 SD = 15.0	Range = 0–40 *M* = 7.5 SD = 13.6	Range = 6–6 *M* = 6 SD = 0

	% Usage^3^				
	L1	L2	L3	L4	L5
Monolinguals	Range = 70–100 *M* = 96.5 SD = 6.0	Range = 0–20 *M* = 4.5 SD = 5.3	Range = 0–9 *M* = 2.3 SD = 4.5	Range = 1–1 *M* = 1.0 SD = 0	*—*
Bilinguals	Range = 5–100 *M* = 66.3 SD = 29.5	Range = 0–90 *M* = 23.8 SD = 23.1	Range = 0–70 *M* = 13.3 SD = 17.8	Range = 0–40 *M* = 6.4 SD = 13.8	Range = 0–0 *M* = 0 SD = 0

	Proficiency (from 1 to 7)				
	L1	L2	L3	L4	L5
Monolinguals	Range = 6–7 *M* = 7.0 SD = 0.2	Range = 1–6 *M* = 5.1 SD = 1.2	Range = 1–5 *M* = 2.3 SD = 1.5	Range = 1–3 *M* = 2.0 SD = 1.0	*—*
Bilinguals	Range = 5–7 *M* = 6.8 SD = 0.4	Range = 5–7 *M* = 6.3 SD = 0.8	Range = 1–7 *M* = 4.7 SD = 1.6	Range = 1–7 *M* = 4.0 SD = 2.1	Range = 1–2 *M* = 1.5 SD = 0.7

^1^ Note that one bilingual adult indicated Slovakian as L1 and German as L2. However, since we only invited adults to the study who indicated on the recruitment platform Prolific that their first language was German, and since this was our preregistered criterion, we decided not to exclude this participant from the final sample.

^2^ Note that four participants indicated percentages of exposure to their different languages that did not add up to 100%. The values of these participants were excluded before calculating the descriptive data shown in this table.

^3^ Note that one participant indicated percentages of usage to their different languages that did not add up to 100%. The values of this participant were excluded before calculating the descriptive data shown in this table.

## Appendix B Counterbalancing Information: Retention Trials in the Adult Version

For the adult version of the experiment, we extended the counterbalancing approach of the child version to the higher number of test trials (four in adults vs. two in children). For the retention trials in the adult version, we presented the target and distractor object from a ME disambiguation trial and the target and distractor object from a Pragmatic disambiguation trial. Therefore, we treated the “ergi & sude” and “modi & toma” referent disambiguation trials as groups (one member of them always being in the ME condition and one in the Pragmatic condition) whose objects would be presented together in the retention trials. That is, for example, in an “ergi” or “sude” retention trial, we presented both targets and distractors from their corresponding referent disambiguation trials (i.e., the same objects as for children); and the same applied to the “modi” or “toma” retention trials. Across the 16 experimental versions for adults, we randomized the order of labels, the target locations, and the locations of the other three presented objects. We made sure that each object changed its position from one trial to the next and that within adults, the target locations were fully counterbalanced, such that participants were presented with one target in each of the four locations.

## Appendix C Packages and Functions

For data handling, preparation, and visualization, we used the packages tidyverse version 2.0.0 (Wickham et al. 2019), lubridate version 1.9.2 (Grolemund and Wickham 2011), the function pirateplot() from the package yarrr version 0.1.5 (Phillips 2017), and the function random_id() from the package ids version 1.0.1 (FitzJohn 2017).

For data analysis, we used the following packages and functions: glmer() from the package lme4 version 1.1‐32 (Bates et al. 2015) for GLMMs with binomial error distribution, lmer() from the package lmerTest version 3.1‐3 (Kuznetsova et al. 2017) for LMMs, brm() from the package brms version 2.20.4 (Bürkner 2017) for the multinomial mixed model, and vif() from the package car version 3.1‐2 (Fox and Weisberg 2019) to calculate variance inflation factors.

## Appendix D Additional Analyses

### Analyses Using Continuous Age as Predictor

We preregistered to analyze participants’ correct object choices in disambiguation and retention trials in models that included *continuous age* as a predictor. To ease the model interpretation, we decided to replace this predictor with the factor *age group* and reported the results of this model in the main text. In the following, we report the results of the preregistered models which revealed the same general pattern of results (see Figures D1, D2 and Tables D1, D2).

**TABLE D1:** Model predicting correct choices in referent disambiguation trials by language background, condition, continuous age, their interactions, and gaze order.

	Estimate	SE	*p*	95% CI
Reference groups: monolinguals, ME condition				
Intercept	2.85	0.45	<0.001	2.14, 4.51
Language background	−0.20	0.52	0.696	−1.79, 1.21
Condition	−1.29	0.46	0.005	−2.91, −0.40
Z‐log‐age	0.73	0.33	0.026	0.10, 1.89
Gaze order	−0.26	0.26	0.316	−0.84, 0.26
Language background × condition	−0.16	0.61	0.786	−1.78, 1.56
Language background × z‐log‐age	−0.88	0.48	0.070	−2.79, −0.07
Condition × z‐log‐age	−1.08	0.41	0.008	−2.63, −0.32
Language background × condition × z‐log‐age	0.74	0.59	0.209	−0.41, 2.85
Reference groups: bilinguals, Pragmatic condition				
Intercept	1.20	0.28	<0.001	0.72, 1.85
Language background	0.37	0.33	0.264	−0.33, 1.06
Condition	1.45	0.40	<0.001	0.76, 2.69
Z‐log‐age	−0.49	0.25	0.048	−1.14, −0.05
Gaze order	−0.26	0.26	0.316	−0.84, 0.26
Language background × condition	−0.16	0.61	0.786	−1.78, 1.56
Language background × z‐log‐age	−0.14	0.35	0.701	−0.67, 0.92
Condition × z‐log‐age	0.34	0.43	0.428	−0.90, 1.25
Language background × condition × z‐log‐age	0.74	0.59	0.209	−0.41, 2.85

*Note*: GLMM with binomial error distribution on participants’ correct choices in referent disambiguation trials with language background (monolingual/bilingual), condition (ME/Pragmatic), continuous age (log‐ and z‐transformed; *M* = 0, SD = 1) and all of their interactions as predictors, gaze order (first to target/distractor) as control variable and random intercepts for participants (SD = 0.47). *N*_observations_ = 490. *N*_groups_ = 160. The 95% confidence intervals were obtained via bootstrapping with 1000 boots. The model described the data significantly better than the corresponding null model (*χ* ^2^(7) = 38.04, *p* < 0.001).

**TABLE D2:** Model predicting consistent choices in retention trials by language background, condition, continuous age, their interactions, and gaze order.

	Estimate	SE	*p*	95% CI
Reference groups: monolinguals, ME condition				
Intercept	1.29	0.26	<0.001	0.85, 1.77
Language background	0.45	0.39	0.246	−0.28, 1.29
Condition	−0.15	0.32	0.629	−0.80, 0.49
Z‐log‐age	0.47	0.22	0.034	0.04, 0.92
Language background × condition	−0.16	0.50	0.747	−1.17, 0.81
Language background × z‐log‐age	0.66	0.34	0.053	0.06, 1.36
Condition × z‐log‐age	0.08	0.29	0.776	−0.48, 0.66
Language background × condition × z‐log‐age	−0.24	0.45	0.593	−1.15, 0.63
Reference groups: bilinguals, Pragmatic condition				
Intercept	1.43	0.28	<0.001	0.92, 2.08
Language background	−0.29	0.36	0.427	−1.05, 0.41
Condition	0.31	0.38	0.411	−0.39, 1.10
Z‐log‐age	0.98	0.25	<0.001	0.47, 1.51
Language background × condition	−0.16	0.50	0.746	−1.17, 0.81
Language background × z‐log‐age	−0.42	0.33	0.202	−1.06, 0.27
Condition × z‐log‐age	0.16	0.34	0.644	−0.52, 0.90
Language background × condition × z‐log‐age	−0.24	0.45	0.594	−1.15, 0.61

*Note*: GLMM with binomial error distribution on participants’ consistent choices in retention trials with language background (monolingual/bilingual), condition (ME/Pragmatic), continuous age (log‐ and z‐transformed; *M* = 0, SD = 1) and all of their interactions as predictors, and random intercepts for participants (SD = 0.72). *N*_observations_ = 487. *N*_groups_ = 160. The 95% confidence intervals were obtained via bootstrapping with 1000 boots. The model described the data significantly better than the corresponding null model (*χ* ^2^(7) = 43.25, *p* < 0.001).

### Analyses of All Children Tested Including Those With Less Than 20% Bilingual Input

In the main analyses of participants’ disambiguation and retention performance, we only included those children who matched the criteria for either the mono‐ or bilingual group. However, we tested an additional eight children who did not meet the criteria for either the mono‐ or bilingual group, because they had regular contact with a second language but did not receive at least 20% input in this language. In the following, we report the results of the exploratory analyses including those additional eight children that we categorized here as being bilingual only in a broader sense. These children were exposed to another language, on average, for 5.9% of the time (SD = 4.2, range: 0–10). Specifically, these included children who received only 5% (*n* = 2) or 10% (*n* = 3) input in the additional language(s), children with bilingual input only in their past (*n* = 1), and children who were only exposed to the sound of another language via TV/music (*n* = 1) or one parent talking in another language to family relatives on the phone, but not to the child/other family members (*n* = 1). See Tables D3 (disambiguation) and D4 (retention) for the full model results.

**TABLE D3:** Analysis of correct choices in referent disambiguation trials including bilinguals in a broader sense.

	Estimate	SE	*p*	95% CI
Reference groups = monolinguals, ME condition, children				
Intercept	1.67	0.48	<0.001	0.88, 2.90
Language background	0.43	0.63	0.493	−0.87, 1.96
Condition	0.42	0.65	0.523	−0.99, 2.01
Age group	1.90	0.74	0.010	0.50, 11.18
Gaze order	−0.27	0.26	0.308	−0.81, 0.22
Language background × condition	−0.25	0.92	0.788	−2.49, 1.80
Language background × age group	−1.34	0.96	0.161	−10.89, 0.50
Condition × age group	−2.66	0.92	0.004	−11.64, −0.96
Language background × condition × age group	0.73	1.22	0.547	−2.04, 9.81
Reference groups = bilinguals, Pragmatic condition, adults				
Intercept	0.91	0.30	0.002	0.37, 1.54
Language background	0.42	0.37	0.249	−0.27, 1.17
Condition	1.76	0.47	<0.001	0.90, 2.97
Age group	1.36	0.55	0.014	0.50, 2.86
Gaze order	−0.27	0.26	0.308	−0.81, 0.22
Language background × condition	0.48	0.79	0.541	−1.07, 11.21
Language background × age group	−0.61	0.79	0.440	−2.42, 1.07
Condition × age group	−1.93	0.81	0.017	−3.90, −0.30
Language background × condition × age group	−0.73	1.22	0.547	−11.27, 2.04

*Note*: GLMM with binomial error distribution on children's and adults’ correct choices in referent disambiguation trials with language background (monolingual/bilingual), condition (ME/Pragmatic), age group (children/adults), and all of their interactions as predictors, speaker's gaze order (first to target/distractor) as control variable (reference group = first to distractor), and random intercepts for participants (SD = 0.50). *N*_observations_ = 506. *N*_groups_ = 168. The model bases on all mono‐ and bilinguals, including children categorized as bilingual in a broader sense. The 95% confidence intervals were obtained via bootstrapping with 1000 boots. The model described the data significantly better than the corresponding null model (*χ* ^2^(7) = 38.79, *p* < 0.001).

**TABLE D4:** Analysis of consistent choices in retention trials including bilinguals in a broader sense.

	Estimate	SE	*p*	95% CI
Reference groups = monolinguals, ME condition, children				
Intercept	0.56	0.38	0.142	−0.18, 1.40
Language background	−0.56	0.51	0.269	−1.56, 0.46
Condition	−0.30	0.51	0.551	−1.27, 0.69
Age group	1.08	0.49	0.028	0.12, 2.08
Language background × condition	0.08	0.68	0.902	−1.24, 1.46
Language background × age group	1.27	0.71	0.071	−0.14, 2.66
Condition × age group	0.22	0.65	0.732	−1.11, 1.46
Language background × condition × age group	−0.35	0.93	0.708	−2.04, 1.54
Reference groups = bilinguals, Pragmatic condition, adults				
Intercept	2.01	0.36	<0.001	1.33, 2.87
Language background	−0.45	0.46	0.323	−1.39, 0.47
Condition	0.34	0.48	0.475	−0.60, 1.36
Age group	−2.23	0.51	<0.001	−3.29, −1.31
Language background × condition	−0.26	0.63	0.674	−1.59, 0.99
Language background × age group	0.93	0.69	0.177	−0.40, 2.23
Condition × age group	−0.13	0.67	0.849	−1.42, 1.19
Language background × condition × age group	0.35	0.93	0.708	−1.54, 2.04

*Note*: GLMM with binomial error distribution on children's and adults’ consistent choices in retention trials with language background (monolingual/bilingual), condition (ME/Pragmatic), age group (children/adults) and all of their interactions as predictors, and random intercepts for participants (SD = 0.71). *N*_observations_ = 503. *N*_groups_ = 168. The model bases on all mono‐ and bilinguals, including children categorized as bilingual in a broader sense. The 95% confidence intervals were obtained via bootstrapping with 1000 boots. The model described the data significantly better than the corresponding null model (*χ* ^2^(7) = 50.36, *p* < 0.001).

### Analyses Based on the Original Preregistered Adult Sample

We preregistered to include monolingual adults in the study who indicated that their first language was German and that they were raised with their native language only. Based on these criteria, the majority of the initial monolingual sample (*n* = 27) reported more than 10% exposure to languages other than German, making them less distinct from the bilingual sample. Thus, based on the helpful feedback of an anonymous reviewer, we recruited additional participants and applied stricter monolingualism criteria: In addition to the initial criteria, the new sample indicated that their country of birth was Germany, that they are still living in the country they were born in, and that they were exposed to the German language at least 90% of the time. We recruited new participants until reaching 43 “monolinguals in a stricter sense” who are now included in the main analyses. In the following, we report the results of the analysis based on the initial (preregistered) sample. Note that the pattern of results did not change after changing the inclusion criteria. See Tables D5, D6, D7 and Figure D3 for the full model results based on the preregistered adult sample.

**TABLE D5:** Model on participants' correct choices in referent disambiguation trials based on the preregistered adult sample.

	Estimate	SE	*p*	95% CI
Reference groups: monolinguals, ME condition, children				
Intercept	1.85	0.52	<0.001	1.01, 3.08
Language background	1.00	0.78	0.201	−0.45, 10.44
Condition	0.43	0.66	0.513	−0.90, 2.03
Age group	2.37	0.87	0.006	0.88, 11.74
Gaze order	−0.37	0.28	0.180	−0.93, 0.16
Language background × condition	−1.09	1.03	0.292	−10.32, 0.95
Language background × age group	−2.35	1.15	0.042	−12.33, −0.18
Condition × age group	−3.57	1.03	<0.001	−13.03, −1.91
Language background × condition × age group	2.38	1.37	0.082	−0.12, 12.78
Reference groups: bilinguals, Pragmatic condition, adults				
Intercept	1.03	0.33	0.002	0.46, 1.72
Language background	0.06	0.40	0.884	−0.70, 0.84
Condition	1.84	0.48	<0.001	1.04, 3.08
Age group	1.17	0.59	0.049	0.15, 2.67
Gaze order	−0.37	0.28	0.180	−0.93, 0.16
Language background × condition	1.29	0.90	0.150	−0.22, 11.91
Language background × age group	0.03	0.83	0.968	−1.73, 1.82
Condition × age group	−1.19	0.92	0.197	−3.16, 9.15
Language background × condition × age group	−2.38	1.37	0.082	−13.81, 0.12

*Note*: GLMM with binomial error distribution on participants’ correct choices in referent disambiguation trials with language background (monolingual/bilingual), condition (ME/Pragmatic), age group (children/adults) and all of their interactions as predictors, gaze order (first to target/distractor) as control variable and random intercepts for participants (SD = 0.80). The analysis bases on the preregistered sample of adults (and children). *N*_observations_ = 485. *N*_groups_ = 160. Confidence intervals were obtained via bootstrapping with 1000 boots. The model described the data significantly better than the corresponding null model (*χ* ^2^(7) = 52.77, *p* < 0.001).

**TABLE D6:** Model on adults’ response times in referent disambiguation trials based on the preregistered adult sample.

	Estimate	SE	*p*	95% CI
Reference groups: monolinguals, ME condition				
Intercept	1.96	0.07	<0.001	1.82, 2.10
Language background	−0.07	0.10	0.495	−0.27, 0.13
Condition	0.17	0.06	0.004	0.04, 0.28
Language background × condition	0.06	0.05	0.158	−0.03, 0.15
Z‐age	−0.03	0.04	0.491	−0.11, 0.05
Gaze order	0.03	0.08	0.710	−0.14, 0.20
Reference groups: bilinguals, Pragmatic condition				
Intercept	2.09	0.07	<0.001	1.94, 2.23
Language background	0.04	0.10	0.704	−0.16, 0.24
Condition	−0.20	0.06	<0.001	−0.31, −0.09
Language background × condition	0.06	0.05	0.158	−0.03, 0.15
Z‐age	−0.03	0.04	0.491	−0.11, 0.05
Gaze order	0.03	0.08	0.710	−0.14, 0.20

*Note*: LMM on adults’ log‐transformed response times in referent disambiguation trials with language background (monolingual/bilingual), condition (ME/Pragmatic), and their interaction as predictors, age (z‐transformed; *M* = 0, SD = 1) and gaze order (first to target/distractor) as control variables and random intercepts for participants (SD = 0.37). The analysis bases on the preregistered adult sample. *N*_observations_ = 337. *N*_groups_ = 86. Confidence intervals were obtained via bootstrapping with 1000 boots. The model described the data significantly better than the corresponding null model (*χ* ^2^(3) = 20.25, *p* < 0.001).

**TABLE D7:** Model on participants' consistent choices in retention trials based on the preregistered adult sample.

	Estimate	SE	*p*	95% CI
Reference groups: monolinguals, ME condition, children				
Intercept	0.57	0.39	0.142	−0.18, 1.34
Language background	−0.58	0.55	0.290	−1.69, 0.43
Condition	−0.31	0.51	0.547	−1.39, 0.77
Age group	1.79	0.56	0.001	0.76, 3.10
Language background × condition	0.17	0.73	0.815	−1.28, 1.61
Language background × age group	0.61	0.77	0.432	−1.01, 2.15
Condition × age group	0.42	0.73	0.565	−1.25, 1.97
Language background × condition × age group	−0.63	1.02	0.535	−2.67, 1.59
Reference groups: bilinguals, Pragmatic condition, adults				
Intercept	2.04	0.38	<0.001	1.39, 2.92
Language background	0.43	0.53	0.415	−0.63, 1.59
Condition	0.35	0.48	0.473	−0.70, 1.35
Age group	−2.19	0.55	<0.001	−3.41, −1.22
Language background × condition	−0.46	0.72	0.518	−1.85, 0.98
Language background × age group	−0.03	0.77	0.972	−1.69, 1.66
Condition × age group	−0.21	0.71	0.767	−1.75, 1.26
Language background × condition × age group	0.63	1.02	0.535	−1.59, 2.65

*Note*: GLMM with binomial error distribution on participants’ consistent choices in retention trials with language background (monolingual/bilingual), condition (ME/Pragmatic), age group (children/adults), and all of their interactions as predictors, and random intercepts for participants (SD = 0.79). The analysis bases on the preregistered sample of adults (and children). *N*_observations_ = 481. *N*_groups_ = 159. Confidence intervals were obtained via bootstrapping with 1000 boots. The model described the data significantly better than the corresponding null model (*χ* ^2^(7) = 57.40, *p* < 0.001).
