# Supplementary material for: When Language Background Does Not Matter: Both Mono‐ and Bilingual Children Use Mutual Exclusivity and Pragmatic Context to Learn Novel Words
Source: Dev Sci. 2025 Mar 11;28(3):e13618. doi: 10.1111/desc.13618 (PMC11894922; doi:10.1111/desc.13618)
Supplement: Supplementary file 1 — Supporting Information [file DESC-28-e13618-s001.docx]

# Supplement A Exploratory Analyses: Effects of Continuous Measures of Bilingualism in Adults

In the main analysis, we used only one measure of bilingualism/ language background: whether or not participants were raised bilingual. Thus, we explored whether the alternative, more continuous, measures of bilingualism (number of languages, percentage of L1 exposure, percentage of L1 usage, and their highest proficiency of all their additional languages) predicted differences in their referent disambiguation or retention performance. Therefore, we fitted the main confirmatory models again (correct choices and response times in disambiguation trials, and consistent choices in retention trials): We included only adults’ data, removed the age group predictor and replaced the main bilingualism/language background variable by one of the other bilingualism measures.

Tables S1-S12 show the detailed model results. There were only two significant effects of the alternative bilingualism measures on the outcomes of interest: The higher participants’ L1 exposure (Table S2) and usage (Table S3) was relative to the exposure to/ usage of their additional languages (i.e., the more monolingual with regard to their language exposure/ usage), the better was their performance in the ME disambiguation trials.

**Table S1**

*Effects of Number of Languages on Adults’ Object Choices in Disambiguation Trials*

|  | Estimate | *SE* | *p* | 95% *CI* |
| --- | --- | --- | --- | --- |
| Reference group = ME condition | | | | |
| Intercept | 3.86 | 1.05 | <.001 | 1.90, 6.49 |
| Nr languages | -0.26 | 0.37 | .474 | -1.08, 0.65 |
| Condition | -2.50 | 1.12 | .026 | -5.31, -0.36 |
| Gaze order | -0.52 | 0.31 | .094 | -1.19, 0.08 |
| Nr languages x condition | 0.22 | 0.41 | .593 | -0.75, 1.15 |
| Reference group = Pragmatic condition | | | | |
| Intercept | 1.36 | 0.58 | .019 | 0.27, 2.69 |
| Nr languages | -0.04 | 0.21 | .838 | -0.48, 0.36 |
| Condition | 2.50 | 1.12 | .026 | 0.36, 5.12 |
| Gaze order | -0.52 | 0.31 | .094 | -1.12, 0.10 |
| Nr languages x condition | -0.22 | 0.41 | .593 | -1.09, 0.75 |

*Note.* GLMM with binomial error distribution on adults’ correct choices in referent disambiguation trials with nr languages, condition and their interaction as predictors, speaker’s gaze order as control variable (reference group = first to distractor) and random intercepts for participants (*SD* = 0.53). *N*_observations_= 344. *N*_groups_ = 86. The 95% confidence intervals were obtained via bootstrapping with 1000 boots. The model described the data significantly better than the corresponding null model (*χ*^2^(3) = 34.81, *p* < .001).

**Table S2**

*Effects of L1 Exposure on Adults’ Object Choices in Disambiguation Trials*

|  | Estimate | *SE* | *p* | 95% *CI* |
| --- | --- | --- | --- | --- |
| Reference group = ME condition | | | | |
| Intercept | 1.37 | 0.77 | .076 | 0.02, 3.33 |
| L1 exposure | 0.03 | 0.01 | .017 | 0.00, 0.05 |
| Condition | -0.60 | 0.90 | .506 | -2.86, 1.10 |
| Gaze order | -0.52 | 0.31 | .096 | -1.16, 0.09 |
| L1 exposure x condition | -0.02 | 0.01 | .113 | -0.05, 0.01 |
| Reference group = Pragmatic condition | | | | |
| Intercept | 0.78 | 0.57 | .170 | -0.35, 1.95 |
| L1 exposure | 0.01 | 0.01 | .382 | -0.01, 0.02 |
| Condition | 0.60 | 0.90 | .506 | -1.10, 2.84 |
| Gaze order | -0.52 | 0.31 | .096 | -1.16, 0.09 |
| L1 exposure x condition | 0.02 | 0.01 | .113 | -0.01, 0.05 |

*Note.* GLMM with binomial error distribution on adults’ correct choices in referent disambiguation trials with L1 exposure, condition and their interaction as predictors, speaker’s gaze order as control variable (reference group = first to distractor) and random intercepts for participants (*SD* = 0.38). *N*_observations_= 344. *N*_groups_ = 86. The 95% confidence intervals were obtained via bootstrapping with 1000 boots. The model described the data significantly better than the corresponding null model (*χ*^2^(3) = 40.22, *p* < .001).

**Table S3**

*Effects of L1 Usage on Adults’ Object Choices in Disambiguation Trials*

|  | Estimate | *SE* | *p* | 95% *CI* |
| --- | --- | --- | --- | --- |
| Reference group = ME condition | | | | |
| Intercept | 1.51 | 0.77 | .051 | 0.32, 3.98 |
| L1 usage | 0.02 | 0.01 | .027 | 0.00, 0.05 |
| Condition | -0.95 | 0.91 | .295 | -3.42, -0.68 |
| Gaze order | -0.52 | 0.31 | .095 | -1.24, 0.05 |
| L1 usage x condition | -0.01 | 0.01 | .238 | -0.04, 0.02 |
| Reference group = Pragmatic condition | | | | |
| Intercept | 0.56 | 0.58 | .330 | -0.58, 1.87 |
| L1 usage | 0.01 | 0.01 | .209 | -0.01, 0.02 |
| Condition | 0.95 | 0.91 | .295 | -0.68, 3.42 |
| Gaze order | -0.52 | 0.31 | .095 | -1.24, 0.05 |
| L1 usage x condition | 0.01 | 0.01 | .238 | -0.01, 0.04 |

*Note.* GLMM with binomial error distribution on adults’ correct choices in referent disambiguation trials with L1 usage, condition and their interaction as predictors, speaker’s gaze order as control variable (reference group = first to distractor) and random intercepts for participants (*SD* = 0.40). *N*_observations_= 344. *N*_groups_ = 86. The 95% confidence intervals were obtained via bootstrapping with 1000 boots. The model described the data significantly better than the corresponding null model (*χ*^2^(3) = 39.99, *p* < .001).

**Table S4**

*Effects of Highest Proficiency Amongst Non-L1 Languages on Adults’ Object Choices in Disambiguation Trials*

|  | Estimate | *SE* | *p* | 95% *CI* |
| --- | --- | --- | --- | --- |
| Reference group = ME condition | | | | |
| Intercept | 4.66 | 2.17 | .032 | 1.58, 12.11 |
| Highest proficiency | -0.26 | 0.35 | .453 | -1.40, 0.26 |
| Condition | -3.04 | 2.34 | .193 | -10.43, 0.41 |
| Gaze order | -0.43 | 0.33 | .196 | -1.14, 0.28 |
| Highest proficiency x condition | 0.19 | 0.38 | .502 | -0.43, 1.32 |
| Reference group = Pragmatic condition | | | | |
| Intercept | 1.61 | 1.01 | .108 | -0.29, 3.98 |
| Highest proficiency | -0.07 | 0.16 | .665 | -0.43, 0.26 |
| Condition | 3.04 | 2.34 | .193 | -0.41, 10.70 |
| Gaze order | -0.43 | 0.33 | .196 | -1.14, 0.28 |
| Highest proficiency x condition | -0.19 | 0.38 | .616 | -1.32, 0.43 |

*Note.* GLMM with binomial error distribution on adults’ correct choices in referent disambiguation trials with highest proficiency amongst non-L1 languages, condition and their interaction as predictors, speaker’s gaze order as control variable (reference group = first to distractor) and random intercepts for participants (*SD* = 0.46). *N*_observations_= 304. *N*_groups_ = 76. The 95% confidence intervals were obtained via bootstrapping with 1000 boots. The model described the data significantly better than the corresponding null model (*χ*^2^(3) = 30.09, *p* < .001).

**Table S5**

*Effects of Number of Languages on Adults’ Response Times in Disambiguation Trials*

|  | Estimate | *SE* | *p* | 95% *CI* |
| --- | --- | --- | --- | --- |
| Reference group = ME condition | | | | |
| Intercept | 1.64 | 0.16 | <.001 | 1.33, 1.95 |
| Nr languages | 0.08 | 0.06 | .192 | -0.04, 0.21 |
| Condition | 0.37 | 0.12 | .002 | 0.16, 0.60 |
| z-Age | 0.09 | 0.05 | .085 | -0.01, 0.18 |
| Gaze order | -0.01 | 0.04 | .824 | -0.08, 0.07 |
| Nr languages x condition | -0.07 | 0.05 | .149 | -0.16, 0.01 |
| Reference group = Pragmatic condition | | | | |
| Intercept | 2.01 | 0.16 | <.001 | 1.70, 2.32 |
| Nr languages | 0.01 | 0.06 | .833 | -0.10, 0.13 |
| Condition | -0.37 | 0.12 | .002 | -0.60, -0.16 |
| z-Age | 0.09 | 0.05 | .085 | -0.01, 0.18 |
| Gaze order | -0.01 | 0.04 | .824 | -0.08, 0.07 |
| Nr languages x condition | 0.07 | 0.05 | .149 | -0.01, 0.16 |

*Note.* LMM on adults’ log-transformed response times in referent disambiguation trials with nr languages, condition and their interaction as predictors, z-transformed age and speaker’s gaze order (reference group = first to distractor) as control variables, and random intercepts for participants (*SD* = 0.41). *N*_observations_= 341. *N*_groups_ = 86. The 95% confidence intervals were obtained via bootstrapping with 1000 boots. The model described the data significantly better than the corresponding null model (*χ*^2^(3) = 27.06, *p* < .001).

**Table S6**

*Effects of L1 Exposure on Adults’ Response Times in Disambiguation Trials*

|  | Estimate | *SE* | *p* | 95% *CI* |
| --- | --- | --- | --- | --- |
| Reference group = ME condition | | | | |
| Intercept | 2.01 | 0.17 | <.001 | 1.67, 2.35 |
| L1 exposure | 0.00 | 0.00 | .283 | -0.01, 0.00 |
| Condition | 0.14 | 0.13 | .282 | -0.13, 0.38 |
| z-Age | 0.09 | 0.05 | .086 | -0.01, 0.18 |
| Gaze order | -0.01 | 0.04 | .825 | -0.08, 0.07 |
| L1 exposure x condition | 0.00 | 0.00 | .579 | 0.00, 0.00 |
| Reference group = Pragmatic condition | | | | |
| Intercept | 2.15 | 0.17 | <.001 | 1.81, 2.48 |
| L1 exposure | 0.00 | 0.00 | .515 | -0.01, 0.00 |
| Condition | -0.14 | 0.13 | .282 | -0.38, 0.13 |
| z-Age | 0.09 | 0.05 | .086 | -0.01, 0.18 |
| Gaze order | -0.01 | 0.04 | .825 | -0.08, 0.07 |
| L1 exposure x condition | 0.00 | 0.00 | .579 | 0.00, 0.00 |

*Note.* LMM on adults’ log-transformed response times in referent disambiguation trials with L1 exposure, condition and their interaction as predictors, z-transformed age and speaker’s gaze order (reference group = first to distractor) as control variables, and random intercepts for participants (*SD* = 0.41). *N*_observations_= 341. *N*_groups_ = 86. The 95% confidence intervals were obtained via bootstrapping with 1000 boots. The model described the data significantly better than the corresponding null model (*χ*^2^(3) = 25.48, *p* < .001).

**Table S7**

*Effects of L1 Usage on Adults’ Response Times in Disambiguation Trials*

|  | Estimate | *SE* | *p* | 95% *CI* |
| --- | --- | --- | --- | --- |
| Reference group = ME condition | | | | |
| Intercept | 2.04 | 0.12 | <.001 | 1.70, 2.40 |
| L1 usage | 0.00 | 0.00 | .222 | -0.01, 0.00 |
| Condition | 0.11 | 0.13 | .412 | -0.16, 0.37 |
| z-Age | 0.09 | 0.05 | .085 | -0.01, 0.18 |
| Gaze order | -0.08 | 0.04 | .825 | -0.08, 0.07 |
| L1 usage x condition | 0.00 | 0.00 | .452 | 0.00, 0.00 |
| Reference group = Pragmatic condition | | | | |
| Intercept | 2.15 | 0.18 | <.001 | 1.81, 2.48 |
| L1 usage | 0.00 | 0.00 | .517 | -0.01, 0.00 |
| Condition | -0.11 | 0.13 | .412 | -0.37, 0.16 |
| z-Age | 0.09 | 0.05 | .085 | -0.01, 0.18 |
| Gaze order | -0.01 | 0.04 | .825 | -0.08, 0.07 |
| L1 usage x condition | 0.00 | 0.00 | .452 | 0.00, 0.00 |

*Note.* LMM on adults’ log-transformed response times in referent disambiguation trials with L1 usage, condition and their interaction as predictors, z-transformed age and speaker’s gaze order (reference group = first to distractor) as control variables, and random intercepts for participants (*SD* = 0.41). *N*_observations_= 341. *N*_groups_ = 86. The 95% confidence intervals were obtained via bootstrapping with 1000 boots. The model described the data significantly better than the corresponding null model (*χ*^2^(3) = 25.89, *p* < .001).

**Table S8**

*Effects of Highest Proficiency Amongst Non-L1 Languages on Adults’ Response Times in Disambiguation Trials*

|  | Estimate | *SE* | *p* | 95% *CI* |
| --- | --- | --- | --- | --- |
| Reference group = ME condition | | | | |
| Intercept | 1.51 | 0.30 | <.001 | 0.93, 2.07 |
| Highest proficiency | 0.05 | 0.05 | .283 | -0.04, 0.15 |
| Condition | 0.31 | 0.22 | .166 | -0.14, 0.73 |
| z-Age | 0.08 | 0.06 | .184 | -0.043 0.19 |
| Gaze order | 0.01 | 0.04 | .865 | -0.08, 0.10 |
| Highest proficiency x condition | -0.02 | 0.04 | .584 | -0.09, 0.05 |
| Reference group = Pragmatic condition | | | | |
| Intercept | 1.82 | 0.30 | <.001 | 1.21, 2.41 |
| Highest proficiency | 0.03 | 0.05 | .506 | -0.06, 0.13 |
| Condition | -0.31 | 0.22 | .166 | -0.73, 0.14 |
| z-Age | 0.08 | 0.06 | .184 | -0.03, 0.19 |
| Gaze order | 0.01 | 0.04 | .865 | -0.08, 0.10 |
| Highest proficiency x condition | 20.01 | 0.04 | .584 | -0.05, 0.09 |

*Note.* LMM on adults’ log-transformed response times in referent disambiguation trials with highest proficiency amongst non-L1 languages, condition and their interaction as predictors, z-transformed age and speaker’s gaze order (reference group = first to distractor) as control variables, and random intercepts for participants (*SD* = 0.43). *N*_observations_= 301. *N*_groups_ = 76. The 95% confidence intervals were obtained via bootstrapping with 1000 boots. The model described the data significantly better than the corresponding null model (*χ*^2^(3) = 18.40, *p* < .001).

**Table S9**

*Effects of Number of Languages on Adults’ Object Choices in Retention Trials*

|  | Estimate | *SE* | *p* | 95% *CI* |
| --- | --- | --- | --- | --- |
| Reference group = ME condition | | | | |
| Intercept | 2.04 | 0.76 | .007 | 0.71, 3.71 |
| Nr languages | 0.02 | 0.29 | .941 | -0.53, 0.65 |
| Condition | -0.72 | 0.91 | .428 | -2.71, 1.00 |
| Nr languages x condition | 0.22 | 0.36 | .538 | -0.51, 1.01 |
| Reference group = Pragmatic condition | | | | |
| Intercept | 1.31 | 0.72 | .067 | -0.14, 2.95 |
| Nr languages | 0.25 | 0.29 | .391 | -0.29, 0.90 |
| Condition | 0.72 | 0.91 | .428 | -1.00, 2.71 |
| Nr languages x condition | -0.22 | 0.36 | .538 | -1.01, 0.51 |

*Note.* GLMM with binomial error distribution on adults’ consistent choices in retention trials with nr languages, condition and their interaction as predictors, and random intercepts for participants (*SD* = 0.97). *N*_observations_= 344. *N*_groups_ = 86. The 95% confidence intervals were obtained via bootstrapping with 1000 boots. The model did not describe the data significantly better than the corresponding null model (*χ*^2^(3) = 1.16, *p* = .763).

**Table S10**

*Effects of L1 Exposure on Adults’ Object Choices in Retention Trials*

|  | Estimate | *SE* | *p* | 95% *CI* |
| --- | --- | --- | --- | --- |
| Reference group = ME condition | | | | |
| Intercept | 2.95 | 0.93 | .002 | 1.43, 5.84 |
| L1 exposure | -0.01 | 0.01 | .307 | -0.04, 0.01 |
| Condition | -0.84 | 1.08 | .440 | -3.77, 1.41 |
| L1 exposure x condition | 0.01 | 0.01 | .535 | -0.02, 0.04 |
| Reference group = Pragmatic condition | | | | |
| Intercept | 2.12 | 0.80 | .008 | 0.72, 4.32 |
| L1 exposure | 0.00 | 0.01 | .756 | -0.03, 0.02 |
| Condition | 0.84 | 1.08 | .440 | -1.41, 3.77 |
| L1 exposure x condition | -0.01 | 0.01 | .535 | -0.04, 0.02 |

*Note.* GLMM with binomial error distribution on adults’ consistent choices in retention trials with nr languages, condition and their interaction as predictors, and random intercepts for participants (*SD* = 0.96). *N*_observations_= 344. *N*_groups_ = 86. The 95% confidence intervals were obtained via bootstrapping with 1000 boots. The model did not describe the data significantly better than the corresponding null model (*χ*^2^(3) = 1.52, *p* = .678).

**Table S11**

*Effects of L1 Usage on Adults’ Object Choices in Retention Trials*

|  | Estimate | *SE* | *p* | 95% *CI* |
| --- | --- | --- | --- | --- |
| Reference group = ME condition | | | | |
| Intercept | 2.58 | 0.93 | .005 | 1.09, 5.84 |
| L1 usage | -0.01 | 0.01 | .565 | -0.04, 0.01 |
| Condition | -0.56 | 1.09 | .611 | -3.62, 1.92 |
| L1 usage x condition | 0.00 | 0.01 | .731 | -0.02, 0.04 |
| Reference group = Pragmatic condition | | | | |
| Intercept | 2.03 | 0.83 | .015 | 0.64, 4.46 |
| L1 usage | 0.00 | 0.01 | .862 | -0.03, 0.02 |
| Condition | 0.56 | 1.09 | .611 | -1.92, 3.62 |
| L1 usage x condition | 0.00 | 0.01 | .731 | -0.04, 0.02 |

*Note.* GLMM with binomial error distribution on adults’ consistent choices in retention trials with L1 usage, condition and their interaction as predictors, and random intercepts for participants (*SD* = 0.96). *N*_observations_= 344. *N*_groups_ = 86. The 95% confidence intervals were obtained via bootstrapping with 1000 boots. The model did not describe the data significantly better than the corresponding null model (*χ*^2^(3) = 0.74, *p* = .863).

**Table S12**

*Effects of Highest Proficiency Amongst Non-L1 Languages on Adults’ Object Choices in Retention Trials*

|  | Estimate | *SE* | *p* | 95% *CI* |
| --- | --- | --- | --- | --- |
| Reference group = ME condition | | | | |
| Intercept | 1.76 | 1.46 | .228 | -0.92, 5.68 |
| Highest proficiency | 0.09 | 0.25 | .717 | -0.51, 0.57 |
| Condition | 1.11 | 1.86 | .553 | -2.89, 4.96 |
| Highest proficiency x condition | -0.21 | 0.31 | .502 | -0.84, 0.46 |
| Reference group = Pragmatic condition | | | | |
| Intercept | 2.87 | 1.60 | .072 | 0.10, 7.25 |
| Highest proficiency | -0.12 | 0.26 | .640 | -0.79, 0.37 |
| Condition | -1.11 | 1.86 | .553 | -4.96, 2.89 |
| Highest proficiency x condition | 0.21 | 0.31 | .502 | -0.46, 0.84 |

*Note.* GLMM with binomial error distribution on adults’ consistent choices in retention trials with highest proficiency amongst non-L1 languages, condition and their interaction as predictors, and random intercepts for participants (*SD* = 1.15). *N*_observations_= 304. *N*_groups_ = 76. The 95% confidence intervals were obtained via bootstrapping with 1000 boots. The model did not describe the data significantly better than the corresponding null model (*χ*^2^(3) = 0.58, *p* = .900).
